# Highly Heterogeneous Soil Bacterial Communities around Terra Nova Bay of Northern Victoria Land, Antarctica

**DOI:** 10.1371/journal.pone.0119966

**Published:** 2015-03-23

**Authors:** Mincheol Kim, Ahnna Cho, Hyoun Soo Lim, Soon Gyu Hong, Ji Hee Kim, Joohan Lee, Taejin Choi, Tae Seok Ahn, Ok-Sun Kim

**Affiliations:** 1 Arctic Research Center, Korea Polar Research Institute, Incheon, Republic of Korea; 2 Division of Life Sciences, Korea Polar Research Institute, Incheon, Republic of Korea; 3 Department of Environmental Science, Kangwon National University, Chuncheon, Republic of Korea; 4 Department of Geological Sciences, Pusan National University, Busan, Republic of Korea; 5 Department of New Antarctic Station, Korea Polar Research Institute, Incheon, Republic of Korea; 6 Division of Climate Change, Korea Polar Research Institute, Incheon, Republic of Korea; Argonne National Laboratory, UNITED STATES

## Abstract

Given the diminished role of biotic interactions in soils of continental Antarctica, abiotic factors are believed to play a dominant role in structuring of microbial communities. However, many ice-free regions remain unexplored, and it is unclear which environmental gradients are primarily responsible for the variations among bacterial communities. In this study, we investigated the soil bacterial community around Terra Nova Bay of Victoria Land by pyrosequencing and determined which environmental variables govern the bacterial community structure at the local scale. Six bacterial phyla, *Actinobacteria*, *Proteobacteria*, *Acidobacteria*, *Chloroflexi*, *Cyanobacteria*, and *Bacteroidetes*, were dominant, but their relative abundance varied greatly across locations. Bacterial community structures were affected little by spatial distance, but structured more strongly by site, which was in accordance with the soil physicochemical compositions. At both the phylum and species levels, bacterial community structure was explained primarily by pH and water content, while certain earth elements and trace metals also played important roles in shaping community variation. The higher heterogeneity of the bacterial community structure found at this site indicates how soil bacterial communities have adapted to different compositions of edaphic variables under extreme environmental conditions. Taken together, these findings greatly advance our understanding of the adaption of soil bacterial populations to this harsh environment.

## Introduction

Most of Antarctica is covered by ice sheets and snow, with only 0.32% of the continent remaining seasonally ice- and snow-free [1]. The ice-free regions are patchily distributed across the continent, and primarily confined to coastal margins. The rest of the ice-free regions are found in deserts, isolated nunataks, and mountain peaks. Victoria Land is one of the largest ice-free regions in continental Antarctica, covering the latitudinal gradient of 8° from Darwin Glacier to Cape Adare. The majority of ice-free areas in this region are found across the Transantarctic Mountains and low-elevation coastal areas, as well as desert environments in South Victoria Land.

Terrestrial environments in Antarctica were long believed to be sterile habitats devoid of life because of the extreme environmental conditions. Indeed, if any types of organisms were found, the level of biodiversity was expected to be low, with populations dominated by a simpler food-web system. However, recent molecular studies have revealed that diverse microbes are abundant in soils of many Antarctic regions despite the environmental harshness [2–4]. Bacterial communities of Antarctic soils are known to be highly localized and heterogeneous [3, 5]. Several major bacterial phyla are found consistently across the continent but their relative abundance varies greatly among regions [5]. Given the absence or limited amount of vegetation and small animals in this environment, abiotic factors such as pH, and water content, have been reported as prevailing determinants of microbial community composition and spatial distribution [6, 7]. In addition to the community structure, bacterial diversity, abundance, and functional gene density have also been reported to be affected by environmental conditions to different degrees [8]. Most previous studies of bacterial communities in Antarctic soils have mostly centered on the Peninsula and Southern Victoria Land [5], while soil microbiota of many ice-free regions still remain unexplored. Furthermore, despite the relative importance of abiotic factors in shaping microbial community structure in Antarctica, only limited numbers of environmental variables have been measured and used to identify this possible link with bacterial community structure [7, 9, 10].

In this study, we investigated the soil bacterial community structure in a coastal area of Northern Victoria Land, Terra Nova Bay, and asked the following exploratory questions. (i) What are the dominant bacterial taxa in this area where microscopic taxa have not previously been explored? (ii) What environmental variables predict soil bacterial community structure? (iii) To what extent do topographical differences and spatial distance influence soil bacterial community structure? To answer these questions, bacterial communities at seven different sites were investigated using a 16S rRNA gene-based pyrosequencing approach. A wide variety of environmental variables including earth elements, trace metals, and general climatic and edaphic factors were also measured and related to the bacterial community structure.

## Materials and Methods

### Soil Sampling and Site Description

**Fig 1:**
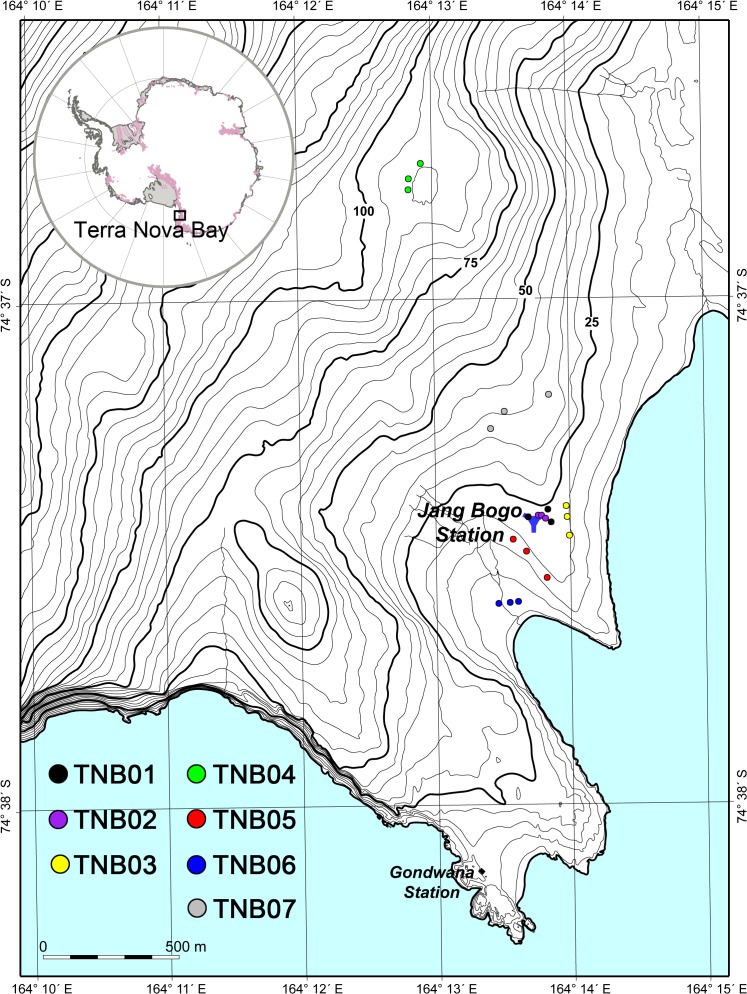
Soil sampling locations in Terra Nova Bay, Antarctica.

Soil samples were collected from Terra Nova Bay (74° 37’ S, 164° 13’ E) in Victoria Land of southeast Antarctica in February 2011 before construction of a new Korean Antarctic research station, Jang Bogo (Fig. 1). Terra Nova Bay is a coastal area positioned in the southernmost edge of North Victoria Land between Cape Washington and the Drygalski Ice Tongue along the east coast. Based on the automatic weather station at the coastal area of Terra Nova Bay (74°37’S, 164° 14’E) that was set up in February of 2010, the mean annual temperature was −14.6°C over 2010–2013, while the annual average wind speed was 4.6 ms^-1^ with predominantly westerly winds. Most of the land gently slopes close to the coast, and this area is mainly composed of exposed bedrock and glacial moraines. Abundant and diverse biota have been reported to inhabit the coastal margin of Terra Nova Bay. A wide variety of life forms including bryophytes, lichens, sea birds, marine mammals, and invertebrates have been observed in this area. A survey of flora and fauna revealed 26 species of lichens (crustose lichens *Buellia* spp. and foliose lichens *Umbilicaria* spp.) and mosses (*Bryum* spp.), and as well as a population of Weddell seals on an ice field and a colony of south polar skua near Gondwana Station of Germany [11]. Soil sampling and field activities in this area were permitted by the Ministry of Foreign Affairs, Republic of Korea. Sampling sites were not located in Antarctic Specially Protected Areas for scientific research in accordance with the Protocol on Environmental Protection to the Antarctic Treaty. In addition, no protected species were sampled in this study.

This area is a formerly glaciated region and a ridge of moraine primarily consisting of unconsolidated glacial debris such as sand and gravel. Coarse-grained mineral soils are frequently found between moderately weathered rocks in most sites. Seven sampling sites were selected based on their topographic or geomorphic differences, a flatland (TNB01) and pond margin (TNB02) near Jang Bogo station, an east-facing hill slope (TNB03), a small lake margin on a hilltop (TNB04), a northwest-facing hill slope (TNB05), an unnamed valley (TNB06), and a south-facing hill slope (TNB07) (S1 Table). Sites are widely spaced (>100m) from one another, being up to 4km apart except for TNB01 and TNB02. Three sampling locations were selected within each site and soil samples of 500g were collected from the surface (0–3 cm, upper layer) and lower soils (3–10 cm, lower layer) at each location using a large sterile spatula. The samples were immediately transported to the laboratory and stored at −80°C until further analysis.

### Physicochemical Analysis of Soil Samples

Water content was determined after drying at 105°C for 48 hours. Using the pH/Cond 340i (WTW GmbH, Germany), pH (1:1 of soil: deionized water slurry) was measured after shaking for 2 h in the dark, while conductivity (1:5 of soil: deionized water slurry) was measured after shaking for 18 h in the dark. Soil samples for grain size analysis were reacted with H_2_O_2_ to remove the organic matter. The size distribution of grains larger than 63 mm (sand and gravel) was determined by dry sieving, while that of the finer grains (silt and clay) was determined by using a Micrometrics Sedigraph 5100. For geochemical analyses of C and N, the powdered samples were dried in an oven at 105°C to remove H_2_O, then cooled at room temperature in desiccators. The total carbon (TC) and nitrogen (TN) contents were analyzed using a FlashEA 1112 elemental analyzer by measuring CO_2_ and NO_2_ generated by combustion at 950°C. The total inorganic carbon (TIC) content was analyzed using a UIC CO_2_ coulometer by measuring the CO_2_ gas generated by the reaction of approximately 50 mg powdered bulk samples with 42.5% phosphoric acid at 80°C for 10 min. The total organic carbon (TOC) content was determined based on the difference between the TC and TIC content. Major elements were analyzed using an X-ray fluorescence spectrometer (XRF; Philips PW244) at the Korea Basic Science Institute (KBSI). Total Fe content was reported as Fe_2_O_3_. Loss on ignition (LOI) was measured by weighing before and after 1 hour calcinations at 1000°C. Trace elements and rare earth elements (REE) concentrations were determined using an inductively coupled plasma atomic emission spectrometer (ICP-AES; Jobin Yvon 138 Ultrace) and inductively coupled plasma mass spectrometer (ICP-MS; Perkin Elmer Elan 6100) at KBSI. The analytical precision for both trace elements and REEs is better than 5%. Major, trace, and REEs concentrations were analyzed three times and averaged (S2 and S3 Tables).

### DNA Extraction, 16S rRNA Gene Pyrosequencing, and Bioinformatic Analysis

Soil DNA was extracted from 0.3g of freeze-dried soils using a FastDNA SPIN Kit (MP Biomedicals, Illkirch, France) according to the manufacturer’s instructions. Extracted soil DNA was amplified using primers targeting the V1−V3 region of the 16S rRNA gene, 27F (5′-AGAGTTTGATCMTGGCTCAG-3′) and 519R (5′-GWATTACCGCGGCKGCTG-3′), as previously described [12]. The PCR conditions were as follows: initial denaturation for 5 min at 94°C, followed by 25 cycles of denaturation (1 min, 94°C), annealing (1 min, 50°C), and extension (1 min 30 sec, 72°C). The PCR mixture consisted of a total volume of 50 μl, 10 pmol of each primer, 1.5U of *Taq* polymerase (GeneAll, Korea), 2/25 volume of dNTP mix, and 1/10 volume of 10x buffer provided with the enzyme. The 454 adapter and barcode sequences for multiplexed sequencing were incorporated into the PCR product. 16S amplicon sequencing was performed by DNALink Inc. (Seoul, Korea) using the 454 GS FLX Titanium Sequencing System (Roche 454 Life Sciences, USA). Raw reads were initially processed using PyroTrimmer, which attempts to trim barcode, linker, and primer sequences and filter out low-quality sequences based on read length, homopolymers, and quality score [13]. The resultant sequences that passed through the initial filtering process were further processed following the 454 SOP using mothur [14]. Each read was taxonomically assigned down to the genus level using the EzTaxon-e database [15]. Raw reads were submitted to the NCBI SRA database with an accession number of SRP041734.

### Statistical Analyses

Read numbers were standardized to 628 per sample to reduce the bias associated with variable pyrosequencing read numbers. Rarefaction curve and diversity indices were generated based on operational taxonomic units (OTUs), which were defined as 97% sequence similarity of the 16S rRNA gene in mothur [14]. A maximum-likelihood (ML) tree was inferred using FastTree2 with the default settings and the resultant tree was used to estimate phylogenetic diversity (Faith’s PD) [16]. Prior to hypothesis testing and statistical analyses, we treated soil samples of the upper and lower layers as replicates of each location because there was no distinct pattern of bacterial diversity or community structure observed between samples from the two soil depths. A Kruskal−Wallis test was performed to determine if there were any significant differences in diversity levels among the seven site categories. Bray−Curtis dissimilarities were calculated using the hellinger-transformed OTU matrix and untransformed phylum abundance matrix. Non-metric multidimensional scaling (NMDS) analysis was conducted using the two dissimilarity matrices at both the phylum and OTU levels. Each environmental variable was fitted onto the ordination space using the ‘envfit’ function in the vegan R package, and the significance of each correlation was tested based on 999 permutations [17]. We employed distance-based redundancy analysis (dbRDA) for constrained ordination using the ‘capscale’ function in vegan. First, skewed variables were normalized according to the ‘draftsman plot’ result in PRIMER v6 [18]. Temperature, water content, pH, Al_2_O_3_, K_2_O, Na_2_O, and SiO_2_ remained untransformed, while the rest were log(x+1) transformed. Values below the analytical detection limits were set to half the value of the detection limit. Multicollinearity between variables was tested using Spearman’s rank correlation in ‘varclus’ function in the nmle R package [19]. Highly correlated variables (Spearman’s ρ^2^>0.70) were excluded, resulting in 35 out of 65 variables being retained (S1 Fig.). Forward model selection based on the Akaike information criteria (AIC) [20] was used to identify the set of environmental variables that best explained the community variation. To determine if bacterial community composition differed significantly between sites, we used two different statistical methods. Specifically, a permutational multivariate analysis of variance (PERMANOVA) [21] was performed with 999 permutations using the ‘adonis’ function in vegan, and analysis of similarity (ANOSIM) implemented in PRIMER v6 was employed to identify any differences between sites (global test) together with pairwise comparisons.

Previously transformed physicochemical variables were normalized to zero mean and unit variance, after which the Euclidean distances between those normalized variables were calculated. The relative similarity of environmental variables between soil samples was visualized using NMDS and the significance of site-specific differences in soil characteristics was tested using PERMANOVA and ANOSIM as described above. To assess the effect of spatial variance between sampling points, the spatial structure between soil samples was modeled using Principal Coordinates of Neighbor Matrices (PCNM) with the ‘quickPCNM’ function in the PCNM R package. A partial Mantel test was performed between the Bray-Curtis dissimilarity of bacterial communities and Euclidean distance of soil properties controlling for geographic distance between locations. The congruence between NMDS plots derived from those matrices was further visualized using Procrustes analysis in vegan and the significance of the Procrustes statistic (Procrustes sum of squares, *m*_12_^2^) was tested by 999 permutations. All statistical analyses were performed using versatile packages in R version 3.0.2 (www.r-project.org).

## Results

### Soil Physicochemical Characteristics and Sample Clustering by Environmental Variables

**Fig 2:**
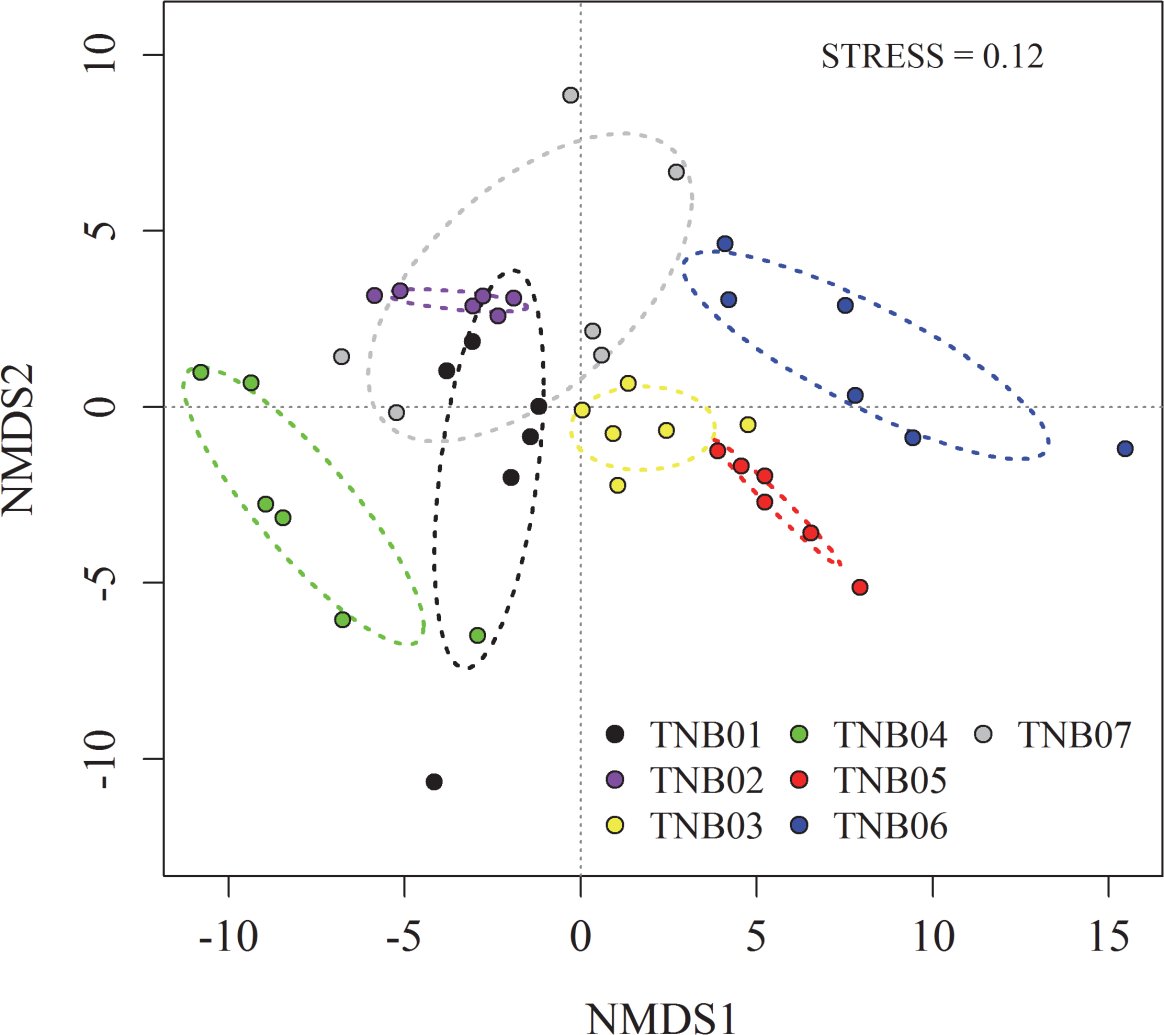
Clustering of soil samples based on environmental variations.

Soil particles were mainly composed of sand (54.6% on average), and to a lesser extent gravel (28.5%) and silt (10.3%) across all samples, suggesting that the dominant soil type of this area is gravelly muddy sand according to the soil classification by Folk, Andrews and Lewis [22]. Very low levels of carbon and nitrogen were observed in the study area (TC 0.36±0.25%, TOC 0.34±0.25%, and TN 0.02±0.02%). The major element compositions varied considerably across soil samples and a broader range of environmental gradients was found in certain variables (S2 and S3 Tables). Soil pH ranged from moderately acidic (5.19) to highly alkaline (9.27). Water content varied considerably among sites, from 1.28% to 19.28%. Conductivity also showed a broad range of variation from 10 to 908 μS cm^-1^. Pearson correlation analysis revealed that certain variables were highly correlated with other variables. Higher water contents were found in soils with higher silt (*r* = 0.73), Al_2_O_3_ (*r* = 0.74), and P_2_O_5_ contents (*r* = 0.73). SiO_2_ was negatively correlated with many other variables, including some major elements (Al_2_O_3_, Fe_2_O_3_, MnO, TiO_2_) and trace elements (Zn, Sc, Be, Ga, Rb) (all *r*<−0.80). The physicochemical properties of soils differed significantly between sampling sites, suggesting that soil samples within a site share more similar chemical compositions (PERMANOVA, pseudo-*F*_6,35_ = 9.37, *P*<0.001; ANOSIM, Global *R* = 0.66, *P*<0.001) (Fig. 2). Samples from site TNB04, which was a small lake margin on a hilltop, were most unique in terms of physicochemical properties (ANOSIM pairwise test, 0.59<*R*<0.98 and all *P*<0.002), while samples from TNB01, a flatland near the station, and TNB07, a south-facing hill slope, had similar soil characteristics (ANOSIM pairwise test, *R* = 0.32, *P*>0.05).

### General Characteristics of Soil Bacterial Communities in Terra Nova Bay

Overall, 55,999 high-quality sequences were retrieved from 42 soil samples (S4 Table). Sequence reads varied across samples from 691 to 2,209. The number of OTUs was similarly variable among samples, ranging from 105 to 312 when estimated based on the standardized read number per sample (n = 628) after removing singleton OTUs, which accounted for 7.1% of the total reads. The highest level of bacterial diversity was observed in a pond margin near the Jang Bogo station (TNB02-BU, Shannon index = 5.47), while the lowest was found in a south-facing hill slope (TNB07-BU, Shannon index = 3.25). The OTU richness and diversity (Shannon, Inverse Simpson, Chao1, and ACE indices) did not differ between sites (Kruskal−Wallis test, all *P*>0.05). The largest diversity range was observed among soil samples of the south-facing hill slope (Shannon index = 3.25−5.36). There was no asymptote in the rarefaction curve, suggesting that bacterial species are far more diverse than expected in this environment (data not shown).

**Fig 3:**
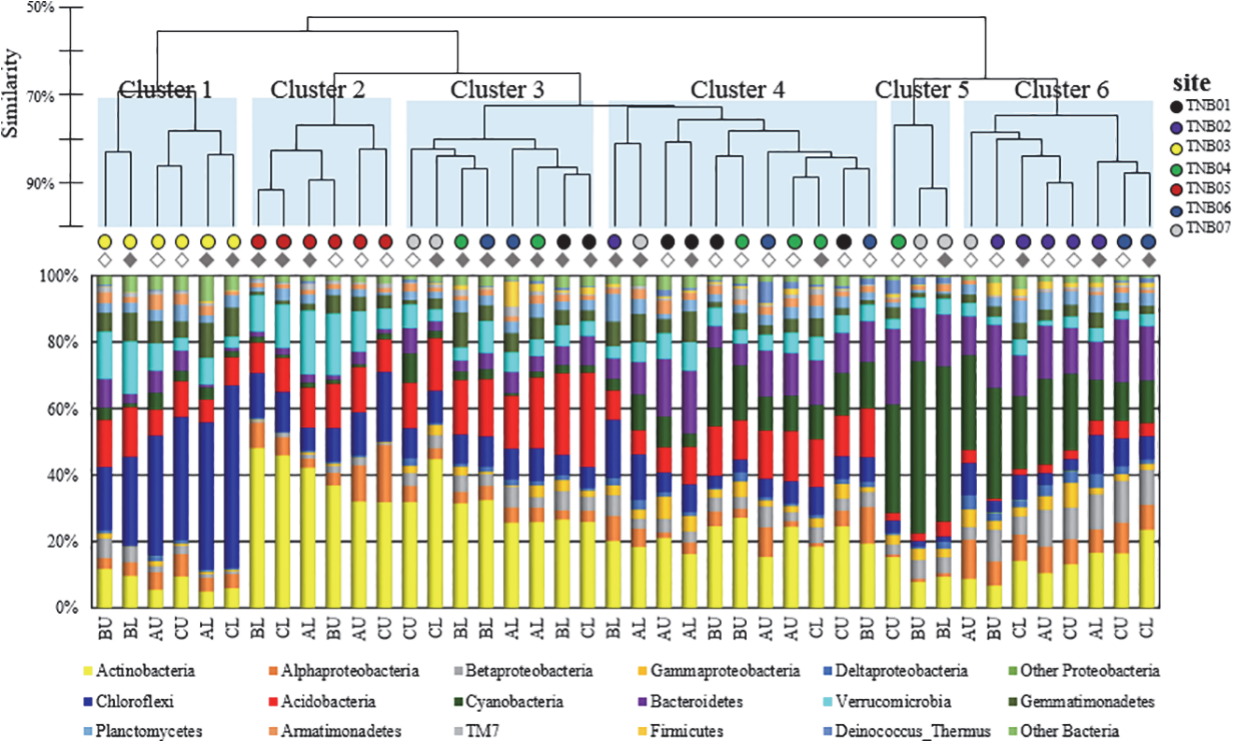
Clustering pattern of soil samples based on bacterial phyla composition. Open and closed circles represent upper and lower soil layers, respectively. The dendrogram on the top was generated using the Bray-Curtis dissimilarity of phyla abundances between samples.

At the phylum level, the soil bacterial community was dominated by six bacterial phyla, *Actinobacteria* (22.5% on average), *Proteobacteria* (14.2%), *Chloroflexi* (11.8%), *Acidobacteria* (11.4%), *Cyanobacteria* (11.2%), and *Bacteroidetes* (8.2%) (Fig. 3). Other phyla such as *Verrucomicrobia* (6.4%), *Gemmatimonadetes* (4.8%), and *Planctomycetes* (3.3%) were also present consistently across all samples, but in lower abundance. The relative abundance of major bacterial phyla varied considerably among samples. Specifically, the largest variation was found in *Cyanobacteria* (0.7–56.1%), followed by *Chloroflexi* (1.4–49.9%), and *Actinobacteria* (5.1–49.5%), and to a lesser extent *Acidobacteria* (0.7–30.0%) and *Proteobacteria* (5.4–28.3%).

### Bacterial Community Structures are Largely Governed by Soil Physicochemical Properties

**Fig 4:**
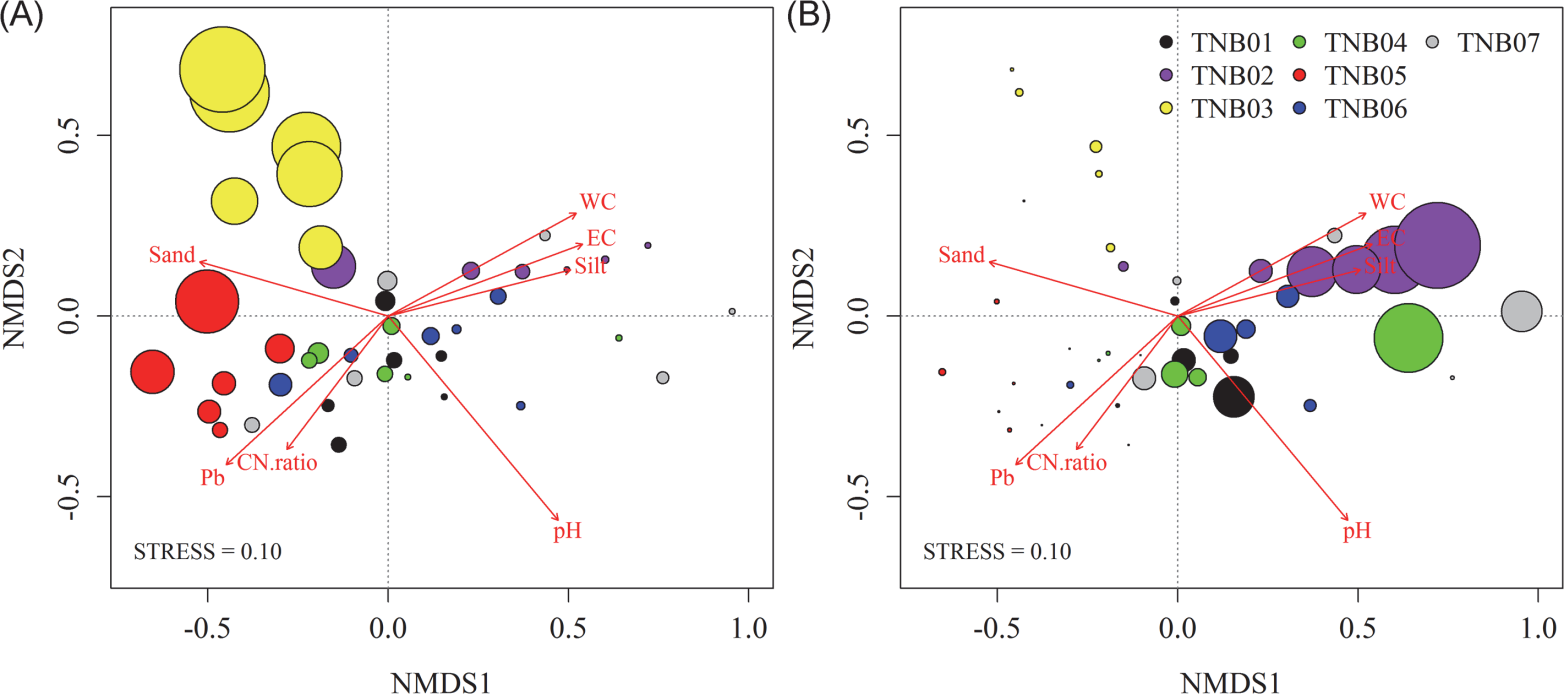
NMDS plot of community composition at the phylum level. Relative abundances of (A) *Chloroflexi* and (B) *Cyanobacteria* are indicated by different-sized circles. Only significant environmental variables were overlaid onto the ordination space.

Phylum composition was moderately but significantly clustered by site (PERMANOVA, pseudo-*F*_6,35_ = 6.42 and *P*<0.001; ANOSIM, Global *R* = 0.50, *P*<0.001), with TNB03 (east-facing hill slope) being the most different from other sites (ANOSIM pairwise test, 0.61<*R*<0.94 and *P*<0.002) (Fig. 4). Given the large variation in relative abundance of bacterial phyla across samples, we expected environmental variables to be major drivers in shaping community structure. Therefore, we investigated which environmental gradients best explained the composition of phyla in soil samples collected from this environment. Four variables showed significant (all *P* <0.01), but moderate correlation with the phylum composition (Fig. 4), pH (*r* ^*2*^ = 0.51), Pb (*r* ^*2*^ = 0.35), water content (*r* ^*2*^ = 0.33), and conductivity (*r* ^*2*^ = 0.31). Sand, silt, and C/N ratio were also significantly correlated, but the association was relatively weak (*r* ^*2*^ = 0.28, 0.26, and 0.20, respectively, all *P* <0.05). When the relative abundance of each phylum was overlaid onto the ordination space, two dominant phyla, *Chloroflexi* and *Cyanobacteria*, showed a clear pattern with environmental variables (Fig. 4). The relative abundance of *Cyanobacteria* was positively correlated with water content, conductivity, and silt content (Pearson’s correlation, *r* = 0.47, 0.47, and 0.48, respectively, all *P*<0.01). *Chloroflexi* abundance was negatively associated with a range of pH 5–9 (*r* = -0.69, *P*<0.01) (S2 Fig.). The negative correlation was also found in major families of *Chloroflexi*. The most dominant class, *Ktedonobacteria*, decreased sharply with increasing pH at a range of pH 5–7 (*r* = −0.66, *P*<0.01), and was almost completely absent from areas with a pH > 7 (S2 Fig.).

**Fig 5:**
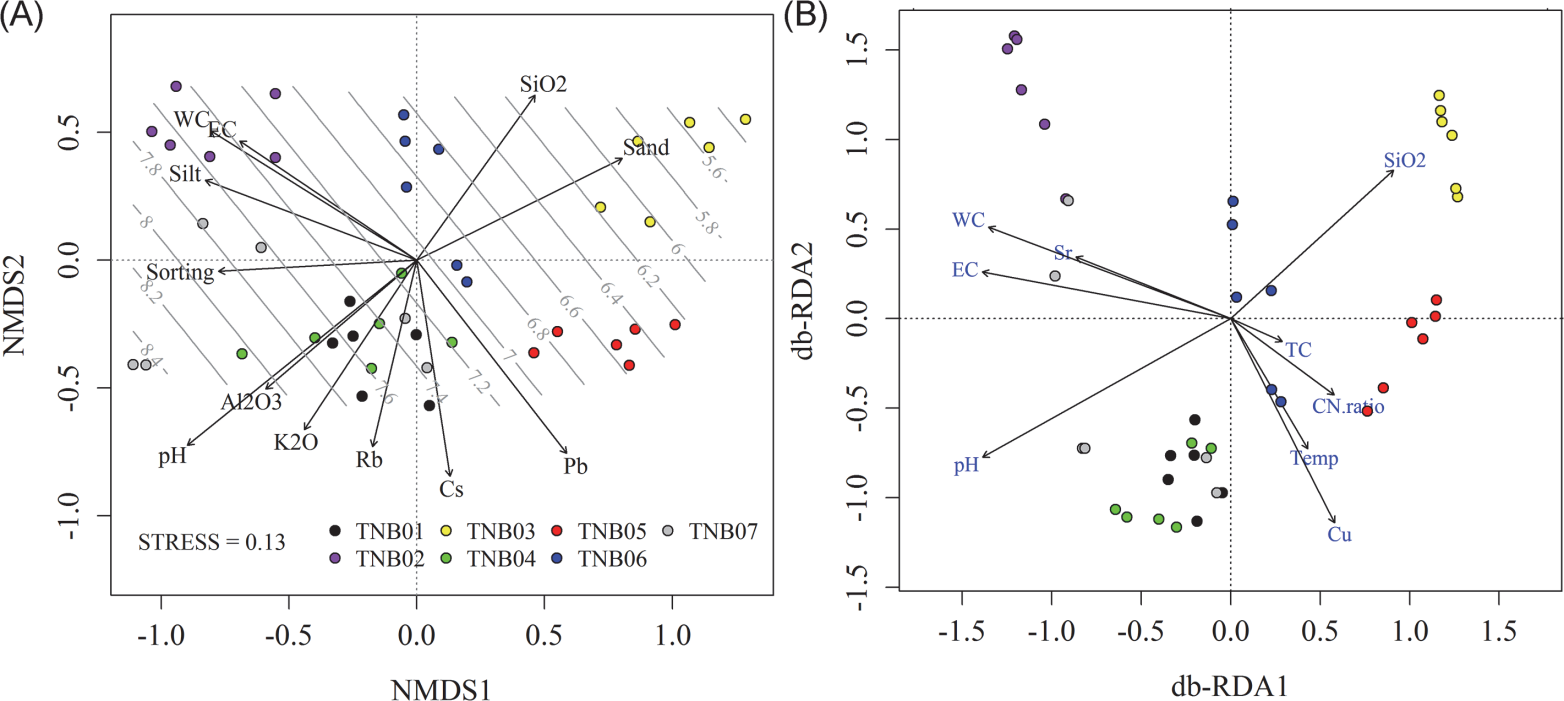
Community composition at the OTU-level. (A) Unconstrained (NMDS) and (B) constrained (db-RDA) ordinations. Highly correlated and significant environmental variables were overlaid onto the NMDS space. Soil pH was linearly correlated with the ordination space using ordisurf.

We also investigated bacterial community clustering at the OTU level, defined here at 97% similarity cutoff of 16S rRNA gene sequences. Similar to the phylum-level results, the OTU-level community structure was clearly grouped by site (PERMANOVA, pseudo-*F*_6,35_ = 4.22 and *P*<0.001; ANOSIM, Global *R* = 0.86, *P*<0.001) (Fig. 5). Pairwise comparison between sites revealed that bacterial communities were unique to each site (ANOSIM pairwise test, 0.59<*R*<1.0, all *P*<0.002), except for those between TNB01 (flatland near the station) and TNB07 (south-facing hill slope) (*R* = 0.35 and *P*>0.05), and between TNB04 (small lake margin on hilltop) and TNB07 (*R* = 0.19 and *P*>0.05). Various variables were found to be significantly correlated with community composition when environmental variables were overlaid onto the ordination space (Fig. 5). pH was the most dominant factor structuring bacterial community in this environment (*r* ^*2*^ = 0.76, *P*<0.001). General chemical variables such as water content and conductivity (*r* ^*2*^ = 0.52, 0.40, all *P*<0.001), soil textual parameters such as sand and silt content (*r* ^*2*^ = 0.46, 0.45, all *P*<0.001), earth elements such as SiO_2_, Al_2_O_3_, and K_2_O (*r* ^*2*^ = 0.36, 0.34, and 0.36, respectively, all *P*<0.001), and trace metals such as Pb and Cs (*r* ^*2*^ = 0.52, 0.42, all *P*<0.001) were also correlated with community composition. Variables strongly correlated with the overall bacterial community were also highly associated with those of certain bacterial phyla (Table 1). Soil pH dominated the community variation of all major bacterial phyla and was more strongly associated with *Actinobacteria* (r = 0.55), while *Cyanobacteria* were least affected by pH (r = 0.32). Notably, *Bacteroidetes* community was moderately, but significantly correlated with conductivity, percent silt, and Pb (r = 0.37, 0.36, and 0.39, respectively).

**Table 1:** Correlations between bacterial community structure and environmental variables.

Phyla	Mantel test based on Spearman’s rank correlation^a^					
	pH	Water content (%)	Conductivity (μS/cm)	% Silt	Pb (ppb)	% Al_2_O_3_
Full community	**0.52**	**0.35**	0.32	0.28	0.29	0.22
Acidobacteria	**0.48**	**0.36**	0.31	0.28	0.22	0.17
Actinobacteria	**0.55**	0.33	**0.35**	0.24	0.20	0.22
Bacteroidetes	**0.37**	**0.37**	**0.37**	**0.36**	**0.39**	0.19
Chloroflexi	**0.46**	**0.35**	0.24	0.25	0.19	0.28
Cyanobacteria	0.32	0.14	0.04	0.05	0.24	0.16
Proteobacteria	**0.41**	0.26	0.19	0.18	0.33	0.20

^a^ Highly correlated variables among all measured soil properties are presented, with significant correlations shown in bold (P<0.01). Phylum-specific bacterial OTU composition (Bray-Curtis dissimilarity) was correlated with each environmental variable using the Mantel test.

In addition to unconstrained ordination, we also conducted constrained ordination (db-RDA) to determine the extent to which bacterial community variation can be explained by environmental variables (Fig. 5). To incorporate spatial trends into the model, we first tested the spatial effects on community structure; however, no significant PCNM variables were generated. Therefore, we only included physicochemical data for the full model. The partial Mantel test also revealed little effect of spatial distance, as indicated by a similar strong correlation between community structure and soil characteristics remaining after removal of the spatial effect (Mantel’s *R* = 0.62 and *P*<0.001; *R* = 0.63 and *P*<0.001 controlling for spatial distance). A strong correlation between environmental variation and bacterial community structure was also confirmed by graphical congruence between two configurations using Procrustes analysis (*m*_12_^2^ = 0.73 and *P*<0.001) (S3 Fig.). Among the 65 environmental variables, 35 were selected based on the rank correlation test between variables. db-RDA showed that almost half of the total variance was explained by the constrained matrix (45.7%). When variables were correlated to each axis, the first axis (db-RDA1) was strongly related to pH (*r* = −0.76), conductivity (*r* = −0.76) and water content (*r* = −0.74), while the second axis (db-RDA2) was best correlated with SiO_2_ (*r* = −0.58).

## Discussion

The overall bacterial community structure in this area did not differ greatly from those found in other areas of Antarctica. However, a large-scale comparison set of variables revealed a strong relationship between the soil bacterial community and edaphic factors. Given the lack of vegetation in this harsh environment, the effects of carbon and nitrogen were almost completely negligible. Instead, other edaphic factors such as pH, water content, and soil texture have more influence on shaping community structure, while earth elements and trace metals also contributed to the community variation.

### Large Variation in Phylum Composition in Terra Nova Bay

Six bacterial phyla, *Actinobacteria*, *Chloroflexi*, *Cyanobacteria*, *Proteobacteria*, *Acidobacteria*, and *Bacteroidetes* were predominant across all soil samples but their relative abundance differed considerably. These phyla were frequently observed in other Antarctic areas, and a large variation in the relative abundance among them depending on regions has also been reported [3, 5]. Among these phyla, a highly abundant feature of *Chloroflexi* in certain samples has not been reported in soils of other Antarctic regions in which their relative abundance was not over 10% of the total bacteria [3]. The relative abundance of *Chloroflexi* was highly variable between sites in this area, ranging from 1.4% to 49.9%. This broader range of *Chloroflexi* abundance may be largely associated with soil pH. There was a strong negative relationship between soil pH and *Chloroflexi* abundance at pH 5–9. Specifically, their abundance decreased sharply from pH 5 to 7, and a lower level of abundance below 10% was maintained from pH 7 to 10. A negative correlation between these factors was also found in a previous study [23]. Interestingly, the relative abundance of four major classes of *Chloroflexi* (*Ktedonobacteria*, HQ190550_c, FR749824_c, and EF018867_c) were also negatively correlated with pH, and they almost completely disappeared in samples with a pH > 7. Most Ktedonobacterial reads were assigned to unknown lineages in the family *Ktedonobacteraceae*. It is currently unclear why the relative abundance of *Chloroflexi* classes was negatively correlated with pH in the range of pH 5–7 and absent under alkaline conditions. Only one species (*Ktedonobacter racemifier*) of this family has been cultured to date, and this filamentous strain is moderately acidophilic, growing well at pH 4.8–6.8 with an optimum pH of 6.0 and no growth observed at ≤ pH 3.9 or ≥ pH 7.5, which is in agreement with the results of the present study [24]. Further physiological tests and metagenome analysis should be conducted to identify functional roles in this environment.

In addition to *Chloroflexi*, other phyla such as *Actinobacteria*, *Cyanobacteria*, and *Acidobacteria* showed large variations in abundance among soils from different sites. The largest variation was found in *Cyanobacteria*, which showed relative abundance values of 0.7% to 56.1%. *Cyanobacteria* have frequently been observed in Antarctic soils with high water contents, and their abundance varies with proximity to water sources [25, 26]. These findings correspond to our finding that the relative abundance of *Cyanobacteria* is positively associated with water content and conductivity. Cyanobacteria were more abundant around pond and valley margins in this study, reflecting the strong association between their abundance and water availability. Less variation was observed in the phylum *Acidobacteria*, which showed relative abundance of 0.7–30.0%. *Acidobacteria* are known to be fastidious and oligotrophic; therefore, they can generally survive under low levels of nutrients and tolerate fluctuations in soil hydration [27]. It is still not clear how these life strategies have enabled their adaptation to the extreme conditions of Antarctic terrestrial environments because their physiology and ecological function are still not well understood.

Interestingly, the majority of acidobacterial reads were confined to Chloracidobacterium_c (subgroup 4) and subgroup 1 and 4 were equally dominant in moderately acidic soils (pH 5.4–6.0). Positive correlation between the abundance of subgroup 4 and soil pH may be a possible explanation as to why this group is dominant in this area [28]. Most subgroup 4 reads were not assigned to known genera owing to the lack of cultured isolates in this group. Some belonged to the genus *Blastocatella* which was recently isolated from semiarid Savana soil [29]. Its broader tolerance of pH (pH 4.0–10.0) may explain its higher abundance at alkaline pH.

### Environmental Variables Have a Greater Effect than Spatial Distance in Shaping Bacterial Community Structure

Soil bacterial communities at this location were predominantly shaped by environmental gradients. Soil pH and water content were good for explaining the variations in bacterial community structure. Soil pH is regarded as one of the best predictors of soil bacterial community structure in various regions worldwide [30–32], including Antarctica [7, 9], which is in accordance with our finding that pH was the dominant factor shaping community structure at this location.

Water content, conductivity, SiO_2_, and trace metals such as Pb and Cu also showed higher correlations with bacterial community structure. In particular, much of the community variation is explained by soil water content in Antarctica. Certain lineages occur more prevalently in drier soils and more *Cyanobacteria*, *Betaproteobacteria*, and *Verrucomicrobia* are found in soils with higher moisture contents [25]. We found a similar pattern, with *Cyanobacteria* being more abundant in higher water content soils, suggesting that water content is a major determinant of cyanobacterial abundance in this environment. Given the lack of vegetation and associated relative dominance of physical weathering, elemental composition of mineral soils has a profound influence on bacterial community structure. Variations in cyanobacterial community are associated with soil elements [29], with Pb content being particularly influential in determining both bacterial and cyanobacterial community compositions [4]. Copper content (Cu) was previously found to be one of the dominant elements shaping bacterial community structure [4, 33]. We also found that soil bacterial community structure was strongly influenced by both Pb and Cu. However, it remains unclear why these elements are strongly associated with the soil bacterial community in this region.

The terrestrial ecosystem of Antarctica shifts rapidly in response to climate change and human activity [34, 35]. Terra Nova Bay surveyed in this study was one of the most undisturbed regions by humans at the time of sampling in 2011. Highly heterogeneous soil bacterial communities were observed between sites, even though they consisted of relatively small areas and were less than 4 km apart. Soil bacterial communities in this pristine area were predominantly structured by environmental gradients and there was little effect of spatial distance. A new Antarctic research station of Korea was just built in this area; accordingly, the area will be subject to anthropogenic impact. The results of the present study will provide an important baseline of information describing how the bacterial population has adapted to the harsh environmental conditions of this area that will be useful in evaluating its responses to changing environmental conditions in the future.

## Supporting Information

S1 Fig Spearman rank correlation between environmental variables. (TIF)

S2 Fig Changing pattern of relative abundance of (A) phylum *Chloroflexi* and (B) class *Ktedonobacteria* with increasing soil pH. (TIF)

S3 Fig Procrustes analysis representing the correlation between bacterial OTU composition (Bray-Curtis dissimilarity) and environmental variation (Euclidean distance). Closed circles represent bacterial OTU composition and closed rectangles indicate environmental variation between samples.
(TIF)

S1 Table Sampling locations and soil physicochemical characteristics. (DOCX)

S2 Table Quantitative analysis of earth element (wt%). (DOCX)

S3 Table Concentration of trace metals in soil samples. (DOCX)

S4 Table Summary of 454 data analysis and diversity estimates. (DOCX)
