# Supplementary figures and images for: Highly Heterogeneous Soil Bacterial Communities around Terra Nova Bay of Northern Victoria Land, Antarctica
Source: PLoS One. 2015 Mar 23;10(3):e0119966. doi: 10.1371/journal.pone.0119966 (PMC4370865; doi:10.1371/journal.pone.0119966)

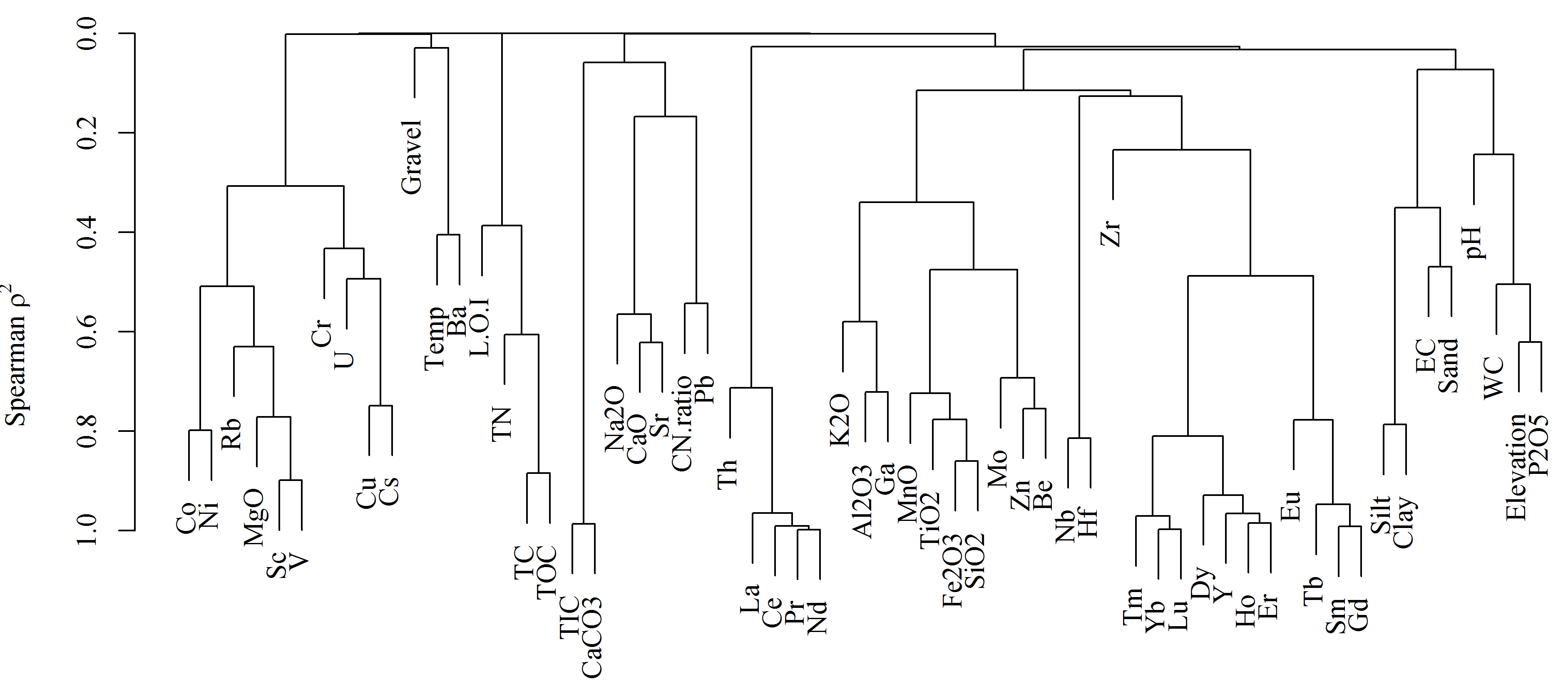

Supplement: S1 Fig — (TIF) [file pone.0119966.s001.tif]

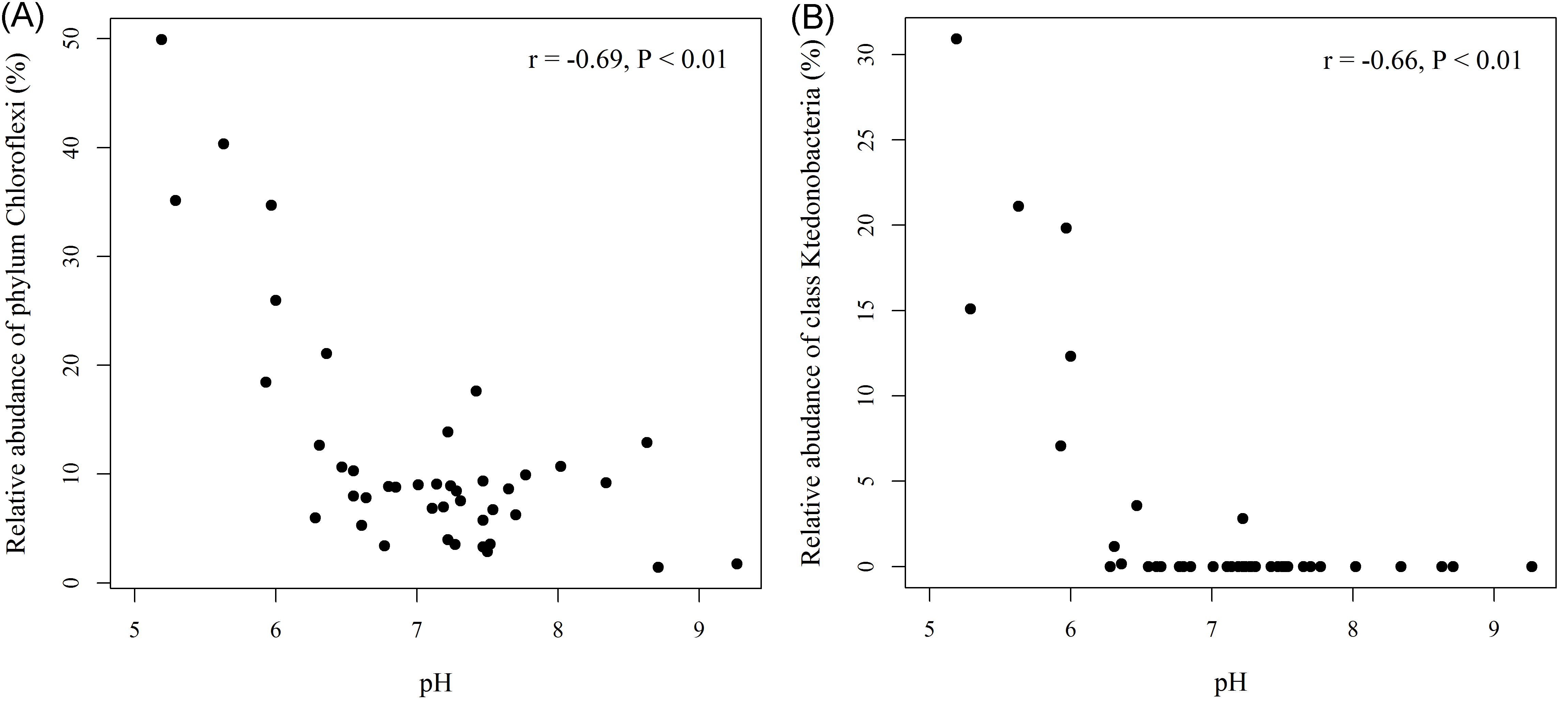

Supplement: S2 Fig — (TIF) [file pone.0119966.s002.tif]

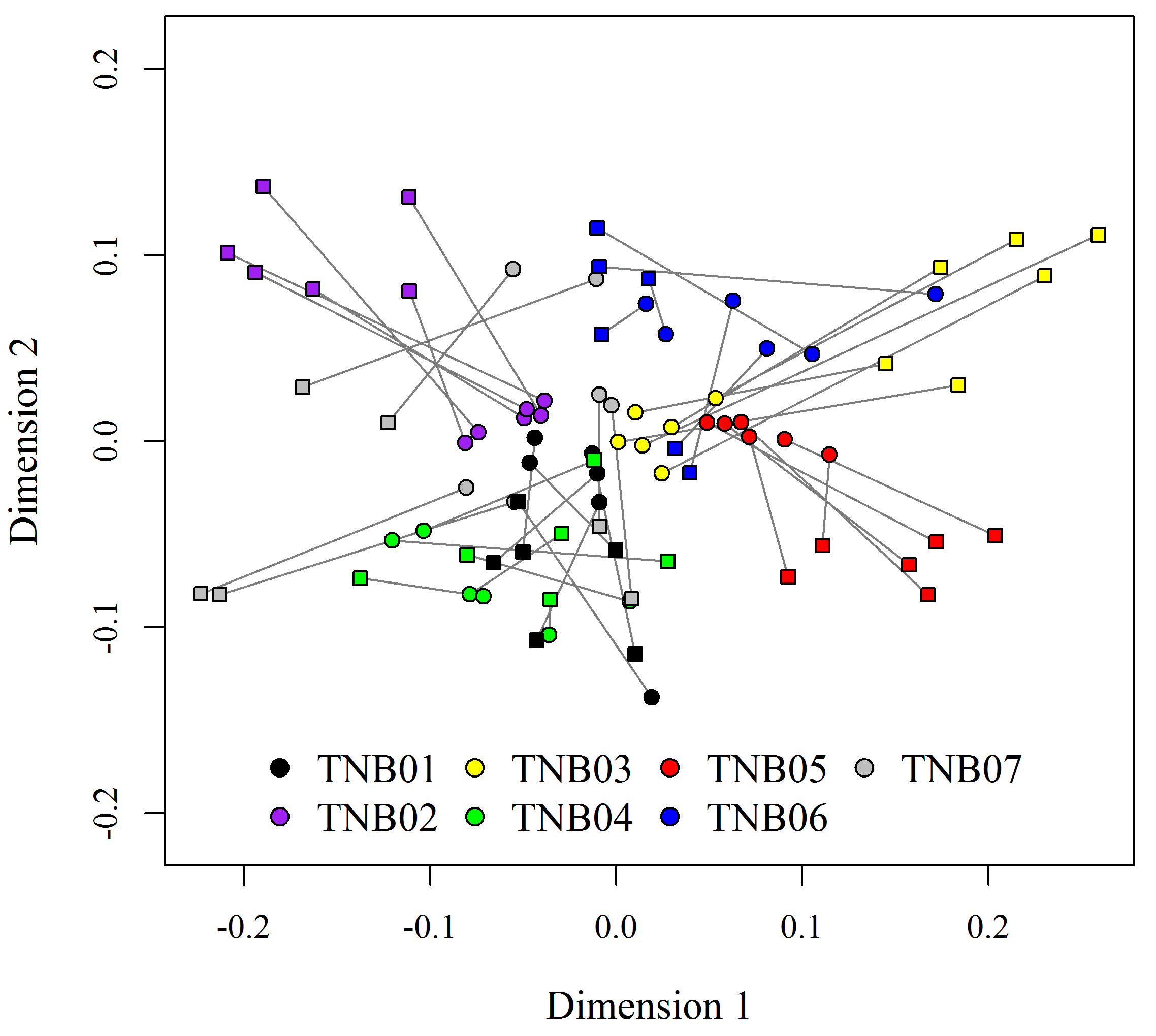

Supplement: S3 Fig — Closed circles represent bacterial OTU composition and closed rectangles indicate environmental variation between samples. (TIF) [file pone.0119966.s003.tif]
