# Supplementary material for: Highly Heterogeneous Soil Bacterial Communities around Terra Nova Bay of Northern Victoria Land, Antarctica
Source: PLoS One. 2015 Mar 23;10(3):e0119966. doi: 10.1371/journal.pone.0119966 (PMC4370865; doi:10.1371/journal.pone.0119966)
Supplement: S1 Table — (DOCX) [file pone.0119966.s004.docx]

**S1 Table. Sampling locations and soil physicochemical characteristics**

| Collection date | Samples name | Latitude  Longitude | Site description | Elevation (m) | Temp  (°C) | Water content  (%) | pH | Con-  ductivity  (µS cm-1) | Composition (%) | | | |  | Textural parameters (φ) | | | | Soil type*** | Chemical properties | | | | | | |
| --- | --- | --- | --- | --- | --- | --- | --- | --- | --- | --- | --- | --- | --- | --- | --- | --- | --- | --- | --- | --- | --- | --- | --- | --- | --- |
|  |  |  |  |  |  |  |  |  | Gravel | Sand | Silt | Clay |  | Mean | Sorting | Skewness | Kurtosis |  | TN  (%) | TC  (%) | TIC  (%) | TOC  (%) | CaCO_3_  (%) | C/N ratio |  |
| February 3rd, 2011 | TNB01-AU* | 74° 37' 25.8" S164° 13' 41.4" E | Flatland near Jang bogo station | 21 | 8.4 | 6.76 | 6.28 | 490 | 31.12 | 27.8 | 22.13 | 18.95 |  | 2.87 | 4.87 | 0.31 | 0.68 | mG | 0.137 | 1.107 | 0.012 | 1.095 | 0.099 | 8.02 |  |
|  | TNB01-AL** |  |  |  |  | 10.85 | 7.24 | 77 | 48.98 | 43.2 | 5.04 | 2.79 |  | -0.07 | 2.63 | 0.49 | 0.78 | msG | 0.085 | 0.737 | 0.015 | 0.722 | 0.122 | 8.49 |  |
|  | TNB01-BU | 74° 37' 24.9" S164° 13' 50.1" E |  | 22 | 6.2 | 5.52 | 7.27 | 79 | 39.19 | 52.62 | 5.25 | 2.93 |  | 0.03 | 2.62 | 0.28 | 0.9 | msG | 0.011 | 0.173 | 0.008 | 0.164 | 0.070 | 14.81 |  |
|  | TNB01-BL |  |  |  |  | 8.16 | 7.47 | 33 | 37.93 | 50.13 | 9.1 | 2.85 |  | 0.25 | 2.82 | 0.29 | 0.95 | msG | 0.005 | 0.154 | 0.01 | 0.144 | 0.085 | 27.69 |  |
|  | TNB01-CU | 74° 37' 26.4" S164° 13' 51.4" E |  | 21 | 3.2 | 7.26 | 7.11 | 161 | 39.7 | 51.52 | 5.46 | 3.32 |  | 0.23 | 2.81 | 0.24 | 0.84 | msG | 0.068 | 0.624 | 0.007 | 0.617 | 0.058 | 9.1 |  |
|  | TNB01-CL |  |  |  |  | 7.74 | 7.70 | 47 | 46.63 | 46.08 | 4.41 | 2.88 |  | -0.17 | 2.69 | 0.33 | 0.88 | msG | 0.029 | 0.395 | 0.011 | 0.384 | 0.094 | 13.06 |  |
|  | TNB02-AU | 74° 37' 25.6" S164° 13' 46.0" E | Unnamed pond margin  Flatland near Jang bogo station | 23 | 2.1 | 19.28 | 6.77 | 557 | 25.92 | 49.38 | 15.47 | 9.23 |  | 1.79 | 4.12 | 0.22 | 1.08 | gmS | 0.051 | 0.512 | 0.015 | 0.497 | 0.127 | 9.7 |  |
|  | TNB02-AL |  |  |  |  | 11.16 | 8.02 | 101 | 18.21 | 47.42 | 22.48 | 11.89 |  | 2.75 | 4.11 | 0.11 | 0.92 | gmS | 0.007 | 0.141 | 0.017 | 0.124 | 0.142 | 17.7 |  |
|  | TNB02-BU | 74° 37' 25.6" S164° 13' 47.4" E |  | 23 | 0.9 | 16.88 | 7.50 | 308 | 14.36 | 45.94 | 26.45 | 13.25 |  | 3.31 | 3.97 | 0.09 | 0.95 | gmS | 0.018 | 0.25 | 0.015 | 0.235 | 0.127 | 13.28 |  |
|  | TNB02-BL |  |  |  |  | 19.24 | 7.42 | 224 | 5.86 | 44.7 | 37.64 | 11.8 |  | 4.02 | 3.34 | 0.06 | 1.08 | gM | 0.007 | 0.146 | 0.013 | 0.133 | 0.108 | 17.78 |  |
|  | TNB02-CU | 74° 37' 26.0" S164° 13' 49.2" E |  | 23 | 1 | 14.86 | 7.47 | 908 | 22.8 | 50.16 | 17.65 | 9.39 |  | 2.09 | 3.95 | 0.19 | 0.96 | gmS | 0.019 | 0.23 | 0.015 | 0.215 | 0.122 | 11.16 |  |
|  | TNB02-CL |  |  |  |  | 16.81 | 7.31 | 222 | 18.86 | 47.89 | 21.16 | 12.08 |  | 2.71 | 4.09 | 0.16 | 0.87 | gmS | 0.007 | 0.126 | 0.012 | 0.114 | 0.098 | 17.15 |  |
|  | TNB03-AU | 74° 37' 24.5" S164° 13' 58.2" E | East-facing hill slope | 22 | 2 | 4.13 | 5.97 | 51 | 33.99 | 59.64 | 3.56 | 2.81 |  | 0.28 | 2.38 | 0.22 | 0.9 | sG | 0.035 | 0.535 | 0.012 | 0.523 | 0.101 | 15.05 |  |
|  | TNB03-AL |  |  |  |  | 7.61 | 5.63 | 36 | 26.24 | 64.04 | 5.97 | 3.75 |  | 0.73 | 2.68 | 0.12 | 1.1 | gmS | 0.035 | 0.618 | 0.011 | 0.607 | 0.09 | 17.15 |  |
|  | TNB03-BU | 74° 37' 25.8" S164° 13' 58.7" E |  | 20 | 2.6 | 5.7 | 5.93 | 15 | 10.13 | 83.57 | 3.19 | 3.11 |  | 1.28 | 1.94 | 0.02 | 1.29 | gS | 0.027 | 0.452 | 0.01 | 0.442 | 0.084 | 16.48 |  |
|  | TNB03-BL |  |  |  |  | 7.63 | 6.00 | 13 | 14.1 | 74.56 | 5.69 | 5.65 |  | 1.29 | 2.77 | 0.16 | 1.78 | gmS | 0.014 | 0.271 | 0.015 | 0.256 | 0.128 | 18.57 |  |
|  | TNB03-CU | 74° 37' 28.0" S164° 13' 59.5" E |  | 19 | 1.5 | 4.71 | 5.29 | 29 | 26.41 | 67.1 | 2.76 | 3.73 |  | 0.54 | 2.48 | 0.12 | 1.09 | gS | 0.027 | 0.565 | 0.013 | 0.552 | 0.106 | 20.83 |  |
|  | TNB03-CL |  |  |  |  | 8.37 | 5.19 | 22 | 27.3 | 65.79 | 3.17 | 3.74 |  | 0.58 | 2.53 | 0.09 | 1.08 | gS | 0.030 | 0.62 | 0.01 | 0.609 | 0.083 | 20.15 |  |
| February 5th, 2011 | TNB04-AU | 74° 36' 46.9" S164° 12' 49.7" E | Unnamed small lake margin on hilltop | 89 | 3.5 | 7.45 | 7.54 | 130 | 41.4 | 38.35 | 10.83 | 9.43 |  | 1.1 | 4.02 | 0.53 | 1.01 | msG | 0.017 | 1.038 | 0.01 | 1.028 | 0.085 | 61.81 |  |
|  | TNB04-AL |  |  |  |  | 10.54 | 7.77 | 59 | 29.99 | 41.53 | 14.53 | 13.95 |  | 2.03 | 4.41 | 0.43 | 0.75 | gmS | 0.013 | 0.943 | 0.01 | 0.934 | 0.08 | 73.35 |  |
|  | TNB04-BU | 74° 36' 45.6" S164° 12' 49.9" E |  | 90 | 5.4 | 14.55 | 7.22 | 66 | 37.72 | 42.28 | 13.48 | 6.52 |  | 1.01 | 3.86 | 0.27 | 0.89 | msG | 0.009 | 0.181 | 0.009 | 0.171 | 0.079 | 18.07 |  |
|  | TNB04-BL |  |  |  |  | 13.53 | 7.14 | 37 | 30.88 | 44.7 | 14.25 | 10.17 |  | 1.86 | 4.03 | 0.2 | 0.83 | msG | 0.008 | 0.208 | 0.013 | 0.195 | 0.105 | 24.12 |  |
|  | TNB04-CU | 74° 36' 43.8" S164° 12' 55.3" E |  | 90 | 6.9 | 10.53 | 7.52 | 222 | 11.2 | 39.12 | 32.2 | 17.48 |  | 4 | 4.06 | 0.01 | 0.93 | gM | 0.015 | 0.324 | 0.008 | 0.317 | 0.064 | 20.68 |  |
|  | TNB04-CL |  |  |  |  | 11.83 | 7.65 | 56 | 38.35 | 55.59 | 3.96 | 2.11 |  | 0.15 | 2.48 | 0.12 | 0.84 | sG | 0.011 | 0.276 | 0.009 | 0.268 | 0.075 | 24.96 |  |
| February 11th, 2011 | TNB05-AU | 74° 37' 28.4" S164° 13' 34.4" E | Northwest-facinghill slope | 13 | 5.5 | 3.61 | 7.22 | 35 | 38.22 | 60.31 | 0.81 | 0.66 |  | -0.14 | 1.81 | 0.18 | 0.84 | sG | 0.014 | 0.319 | 0.008 | 0.311 | 0.067 | 22.61 |  |
|  | TNB05-AL |  |  |  |  | 3.26 | 7.19 | 21 | 32.66 | 65.74 | 0.87 | 0.74 |  | -0.05 | 1.77 | 0.05 | 0.94 | sG | 0.006 | 0.285 | 0.008 | 0.278 | 0.064 | 43.87 |  |
|  | TNB05-BU | 74° 37' 29.8" S164° 13' 40.5" E |  | 13 | 5.5 | 4.66 | 6.55 | 38 | 26.33 | 68.54 | 2.78 | 2.35 |  | 0.16 | 1.82 | 0.21 | 1.05 | gS | 0.008 | 0.247 | 0.009 | 0.238 | 0.074 | 30.1 |  |
|  | TNB05-BL |  |  |  |  | 4.16 | 6.31 | 30 | 31.67 | 58.98 | 4.84 | 4.51 |  | 0.2 | 2.69 | 0.19 | 1.35 | msG | 0.009 | 0.252 | 0.007 | 0.244 | 0.061 | 28.7 |  |
|  | TNB05-CU | 74° 37' 32.9" S164° 13' 49.3" E |  | 13 | 5.5 | 2.16 | 6.36 | 32 | 25.79 | 68.53 | 3.17 | 2.52 |  | 0.49 | 2.18 | -0.01 | 1.01 | gS | 0.017 | 0.366 | 0.01 | 0.357 | 0.08 | 21.54 |  |
|  | TNB05-CL |  |  |  |  | 3.84 | 6.47 | 23 | 25.36 | 72.82 | 1.13 | 0.68 |  | 0.32 | 1.84 | -0.13 | 0.83 | gS | 0.006 | 0.282 | 0.009 | 0.273 | 0.071 | 42.07 |  |
|  | TNB06-AU | 74° 37' 35.8" S164° 13' 36.6" E | Unnamed valley between Gondwana and Jang Bogo stations | 7 | -3.1 | 1.28 | 6.61 | 18 | 52.1 | 43.15 | 2.12 | 2.63 |  | -0.86 | 2.33 | 0.24 | 0.68 | msG | 0.021 | 0.339 | 0.009 | 0.329 | 0.077 | 15.48 |  |
|  | TNB06-AL |  |  |  |  | 1.76 | 6.80 | 10 | 32.62 | 63.98 | 1.51 | 1.89 |  | 0.08 | 2.18 | -0.23 | 0.77 | sG | 0.005 | 0.114 | 0.009 | 0.105 | 0.076 | 20.85 |  |
|  | TNB06-BU | 74° 37' 35.9" S164° 13' 32.8" E |  | 10 | 0.9 | 1.92 | 6.64 | 23 | 16.72 | 59.09 | 7.29 | 16.9 |  | 3.18 | 4.5 | 0.38 | 1.29 | gmS | 0.008 | 0.17 | 0.011 | 0.159 | 0.089 | 20.26 |  |
|  | TNB06-BL |  |  |  |  | 1.53 | 7.01 | 12 | 18.16 | 78.54 | 1.46 | 1.84 |  | 0.86 | 1.85 | -0.38 | 1.07 | gS | 0.006 | 0.129 | 0.007 | 0.122 | 0.058 | 19.83 |  |
|  | TNB06-CU | 74° 37' 36.0" S164° 13' 27.9" E |  | 8 | 0.9 | 7.37 | 6.85 | 18 | 20.23 | 77.3 | 1.11 | 1.36 |  | 0.12 | 1.75 | -0.23 | 1.05 | gS | 0.008 | 0.151 | 0.009 | 0.142 | 0.077 | 18.31 |  |
|  | TNB06-CL |  |  |  |  | 10.94 | 6.55 | 26 | 21.4 | 75.28 | 1.39 | 1.94 |  | 0.24 | 1.74 | -0.19 | 1.04 | gS | 0.006 | 0.208 | 0.008 | 0.2 | 0.069 | 32.6 |  |
|  | TNB07-AU | 74° 37' 11.4" S164° 13' 51.0" E | South-facing hill slope | 30 | -1.9 | 12.42 | 8.34 | 187 | 43.34 | 42.79 | 9.22 | 4.65 |  | 0.21 | 3.47 | 0.22 | 0.87 | msG | 0.012 | 0.339 | 0.152 | 0.187 | 1.264 | 15.39 |  |
|  | TNB07-AL |  |  |  |  | 15.35 | 8.63 | 96 | 24.9 | 44.74 | 22.51 | 7.84 |  | 2.17 | 4.13 | -0.02 | 0.81 | gmS | 0.007 | 0.264 | 0.181 | 0.083 | 1.507 | 12.17 |  |
|  | TNB07-BU | 74° 37' 13.3" S164° 13' 31.2" E |  | 34 | -1.4 | 10.22 | 9.27 | 234 | 59 | 31.2 | 6.3 | 3.5 |  | -0.93 | 3.25 | 0.66 | 0.88 | msG | 0.027 | 0.258 | 0.007 | 0.252 | 0.056 | 9.28 |  |
|  | TNB07-BL |  |  |  |  | 13.66 | 8.71 | 97 | 8.83 | 50.46 | 29.27 | 11.44 |  | 3.46 | 3.6 | 0.14 | 0.87 | gmS | 0.01 | 0.146 | 0.01 | 0.136 | 0.08 | 13.56 |  |
|  | TNB07-CU | 74° 37' 15.3" S164° 13' 25.1" E |  | 30 | -0.5 | 7.23 | 7.28 | 107 | 24.58 | 67.3 | 5.54 | 2.58 |  | 0.87 | 2.41 | 0.05 | 0.91 | gmS | 0.013 | 0.245 | 0.106 | 0.139 | 0.883 | 10.41 |  |
|  | TNB07-CL |  |  |  |  | 8.23 | 7.47 | 37 | 24.22 | 65.86 | 7.2 | 2.72 |  | 0.93 | 2.62 | -0.01 | 0.97 | gmS | 0.009 | 0.164 | 0.004 | 0.16 | 0.034 | 18.73 |  |
| Min | | |  | 7 | -3.1 | 1.28 | 5.19 | 10 | 5.86 | 27.8 | 0.81 | 0.66 |  | -0.93 | 1.74 | -0.38 | 0.68 |  | 0.005 | 0.114 | 0.004 | 0.083 | 0.034 | 8.02 |  |
| Max | | |  | 90 | 8.4 | 19.28 | 9.27 | 908 | 59 | 83.57 | 37.64 | 18.95 |  | 4.02 | 4.87 | 0.66 | 1.78 |  | 0.137 | 1.107 | 0.181 | 1.095 | 1.507 | 73.35 |  |
| Average | | |  | 29.57 | 2.62 | 8.67 | 6.94 | 134.74 | 28.47 | 54.56 | 10.29 | 6.17 |  | 1.12 | 2.95 | 0.15 | 0.96 |  | 0.02 | 0.36 | 0.02 | 0.34 | 0.2 | 21.99 |  |
| STD | | |  | 26.11 | 3.09 | 4.94 | 0.87 | 174.02 | 12.04 | 13.56 | 9.58 | 5.05 |  | 1.3 | 0.92 | 0.2 | 0.2 |  | 0.02 | 0.25 | 0.04 | 0.25 | 0.3 | 13.21 |  |

* U: upper layer (0-3cm in depth); ** L: lower layer (3-10cmin depth)

***Soil Type: mG, muddy Gravel; msG, muddy sandy Gravel; gmS, gravelly muddy Sand; gM, gravelly Mud; sG, sandy Gravel; gS, gravelly Sand; gM, gravelly Mud
