# Supplementary material for: Highly Heterogeneous Soil Bacterial Communities around Terra Nova Bay of Northern Victoria Land, Antarctica
Source: PLoS One. 2015 Mar 23;10(3):e0119966. doi: 10.1371/journal.pone.0119966 (PMC4370865; doi:10.1371/journal.pone.0119966)
Supplement: S2 Table — (DOCX) [file pone.0119966.s005.docx]

**S2 Table. Quantitative analysis of earth element (wt%)**

| Samples name | Al_2_O_3_ | CaO | Fe_2_O_3_^*^ | K_2_O | MgO | MnO | Na_2_O | P_2_O_5_ | SiO_2_ | TiO_2_ | L.O.I | Total |
| --- | --- | --- | --- | --- | --- | --- | --- | --- | --- | --- | --- | --- |
| TNB01-AU | 12.57 | 5.79 | 5.54 | 2.16 | 3.99 | 0.26 | 2.21 | 0.53 | 63.99 | 0.59 | 3.21 | 101 |
| TNB01-AL | 13.10 | 2.63 | 4.18 | 3.34 | 1.59 | 0.08 | 2.96 | 0.19 | 68.86 | 0.62 | 2.66 | 100 |
| TNB01-BU | 13.13 | 2.89 | 4.23 | 3.25 | 1.57 | 0.08 | 3.28 | 0.17 | 69.69 | 0.61 | 1.38 | 100 |
| TNB01-BL | 13.46 | 2.73 | 4.36 | 3.61 | 1.44 | 0.08 | 3.40 | 0.16 | 69.03 | 0.66 | 1.47 | 100 |
| TNB01-CU | 13.42 | 2.59 | 4.48 | 3.27 | 1.83 | 0.08 | 2.87 | 0.19 | 68.92 | 0.65 | 2.63 | 101 |
| TNB01-CL | 13.55 | 2.53 | 4.14 | 3.20 | 1.56 | 0.08 | 2.97 | 0.17 | 70.85 | 0.62 | 1.99 | 102 |
| TNB02-AU | 12.88 | 2.86 | 3.46 | 3.13 | 1.33 | 0.08 | 3.26 | 0.21 | 69.50 | 0.55 | 2.53 | 99.8 |
| TNB02-AL | 13.40 | 2.87 | 3.97 | 3.29 | 1.34 | 0.09 | 3.35 | 0.22 | 70.60 | 0.61 | 1.88 | 102 |
| TNB02-BU | 13.83 | 2.78 | 4.13 | 3.45 | 1.30 | 0.12 | 3.52 | 0.22 | 68.56 | 0.61 | 2.18 | 101 |
| TNB02-BL | 14.23 | 2.75 | 4.85 | 3.51 | 1.54 | 0.13 | 3.58 | 0.22 | 67.31 | 0.70 | 2.43 | 101 |
| TNB02-CU | 13.90 | 2.84 | 3.86 | 3.37 | 1.25 | 0.09 | 3.62 | 0.22 | 69.62 | 0.60 | 2.17 | 102 |
| TNB02-CL | 14.09 | 2.75 | 4.11 | 3.36 | 1.36 | 0.09 | 3.57 | 0.21 | 69.72 | 0.61 | 1.82 | 102 |
| TNB03-AU | 11.93 | 2.50 | 3.96 | 2.59 | 1.70 | 0.07 | 2.63 | 0.15 | 73.57 | 0.50 | 2.21 | 102 |
| TNB03-AL | 12.18 | 2.59 | 4.51 | 2.51 | 1.85 | 0.08 | 2.46 | 0.19 | 71.82 | 0.58 | 2.59 | 101 |
| TNB03-BU | 12.60 | 2.46 | 3.75 | 2.93 | 1.46 | 0.08 | 2.81 | 0.17 | 71.39 | 0.55 | 1.90 | 100 |
| TNB03-BL | 12.54 | 2.34 | 3.83 | 2.76 | 1.44 | 0.08 | 2.75 | 0.17 | 72.73 | 0.60 | 1.61 | 101 |
| TNB03-CU | 11.04 | 1.91 | 3.57 | 2.73 | 1.17 | 0.06 | 2.43 | 0.16 | 74.15 | 0.46 | 2.36 | 100 |
| TNB03-CL | 11.77 | 2.10 | 3.74 | 2.88 | 1.25 | 0.07 | 2.63 | 0.17 | 72.56 | 0.54 | 2.74 | 100 |
| TNB04-AU | 12.24 | 1.60 | 7.01 | 3.64 | 1.81 | 0.08 | 2.12 | 0.17 | 65.57 | 0.68 | 4.26 | 99 |
| TNB04-AL | 12.56 | 1.47 | 8.58 | 3.76 | 1.91 | 0.10 | 2.09 | 0.18 | 63.67 | 0.78 | 5.46 | 101 |
| TNB04-BU | 14.77 | 3.15 | 6.51 | 3.43 | 2.74 | 0.14 | 3.59 | 0.24 | 64.06 | 0.90 | 1.91 | 101 |
| TNB04-BL | 15.13 | 3.02 | 7.10 | 3.60 | 2.58 | 0.15 | 3.85 | 0.26 | 62.49 | 0.98 | 2.43 | 102 |
| TNB04-CU | 14.57 | 2.48 | 7.57 | 3.91 | 2.85 | 0.12 | 3.24 | 0.22 | 62.29 | 1.00 | 2.92 | 101 |
| TNB04-CL | 14.48 | 2.41 | 7.80 | 3.92 | 2.85 | 0.11 | 2.86 | 0.23 | 62.95 | 1.01 | 3.04 | 102 |
| TNB05-AU | 11.97 | 1.87 | 2.80 | 3.33 | 1.17 | 0.06 | 2.42 | 0.18 | 76.20 | 0.41 | 1.46 | 102 |
| TNB05-AL | 12.05 | 2.20 | 3.46 | 2.75 | 1.44 | 0.07 | 2.45 | 0.14 | 74.06 | 0.51 | 1.44 | 101 |
| TNB05-BU | 12.43 | 2.37 | 3.49 | 2.89 | 1.74 | 0.06 | 2.30 | 0.16 | 74.51 | 0.54 | 1.33 | 102 |
| TNB05-BL | 12.21 | 2.10 | 3.40 | 2.73 | 1.56 | 0.07 | 2.63 | 0.14 | 75.16 | 0.48 | 1.51 | 102 |
| TNB05-CU | 11.72 | 2.08 | 3.34 | 2.75 | 1.43 | 0.06 | 2.56 | 0.15 | 74.43 | 0.48 | 1.65 | 101 |
| TNB05-CL | 11.20 | 1.85 | 3.32 | 3.00 | 1.43 | 0.06 | 2.33 | 0.12 | 73.09 | 0.46 | 1.37 | 98 |
| TNB06-AU | 10.85 | 2.08 | 2.48 | 2.48 | 1.03 | 0.07 | 2.30 | 0.13 | 75.87 | 0.48 | 1.34 | 99 |
| TNB06-AL | 10.69 | 1.92 | 2.14 | 2.60 | 0.91 | 0.06 | 2.34 | 0.12 | 76.74 | 0.42 | 0.74 | 98 |
| TNB06-BU | 10.57 | 2.14 | 2.76 | 2.50 | 1.10 | 0.08 | 2.30 | 0.15 | 75.55 | 0.63 | 1.04 | 98 |
| TNB06-BL | 10.44 | 1.87 | 1.91 | 2.59 | 0.80 | 0.04 | 2.30 | 0.11 | 76.91 | 0.34 | 0.83 | 98 |
| TNB06-CU | 10.08 | 1.68 | 1.58 | 2.44 | 0.69 | 0.03 | 2.18 | 0.09 | 81.67 | 0.23 | 0.86 | 102 |
| TNB06-CL | 10.86 | 1.89 | 2.29 | 2.71 | 0.94 | 0.04 | 2.35 | 0.12 | 77.64 | 0.34 | 1.09 | 100 |
| TNB07-AU | 12.99 | 3.57 | 2.85 | 2.79 | 1.29 | 0.06 | 2.99 | 0.19 | 72.26 | 0.50 | 1.75 | 101 |
| TNB07-AL | 13.00 | 3.82 | 2.93 | 2.78 | 1.12 | 0.06 | 3.03 | 0.22 | 71.73 | 0.55 | 1.70 | 101 |
| TNB07-BU | 13.79 | 3.07 | 5.49 | 3.60 | 2.38 | 0.11 | 3.45 | 0.24 | 64.43 | 0.72 | 1.84 | 99 |
| TNB07-BL | 14.37 | 2.67 | 5.48 | 3.55 | 2.29 | 0.11 | 3.22 | 0.24 | 66.26 | 0.74 | 1.59 | 101 |
| TNB07-CU | 13.48 | 3.05 | 4.45 | 3.31 | 2.10 | 0.09 | 2.93 | 0.18 | 68.22 | 0.65 | 1.48 | 100 |
| TNB07-CL | 12.81 | 3.15 | 4.34 | 3.95 | 1.82 | 0.09 | 3.90 | 0.16 | 66.28 | 0.61 | 1.37 | 98 |
| Min | 10.08 | 1.47 | 1.58 | 2.16 | 0.69 | 0.03 | 2.09 | 0.09 | 62.29 | 0.23 | 0.74 |  |
| Max | 15.13 | 5.79 | 8.58 | 3.95 | 3.99 | 0.26 | 3.90 | 0.53 | 81.67 | 1.01 | 5.46 |  |
| Average | 12.69 | 2.56 | 4.23 | 3.10 | 1.64 | 0.09 | 2.86 | 0.19 | 70.59 | 0.60 | 2.00 |  |
| STD | 1.29 | 0.73 | 1.61 | 0.46 | 0.64 | 0.04 | 0.52 | 0.07 | 4.68 | 0.17 | 0.90 |  |

*Fe_2_O_3_ : Total Fe
