# Supplementary material for: Highly Heterogeneous Soil Bacterial Communities around Terra Nova Bay of Northern Victoria Land, Antarctica
Source: PLoS One. 2015 Mar 23;10(3):e0119966. doi: 10.1371/journal.pone.0119966 (PMC4370865; doi:10.1371/journal.pone.0119966)
Supplement: S3 Table — (DOCX) [file pone.0119966.s006.docx]

**S3 Table. Concentration of trace metals in soil samples.**

| Samples name | Ba | Cr | Sr | Zn | Sc | V | Zr | Be | Co | Ni | Cu | Ga | Rb | Y | Nb | Mo | Cd | Cs | La | Ce | Pr | Nd | Sm | Eu | Gd | Tb | Dy | Ho | Er | Tm | Yb | Lu | Hf | Pb | Th | U |
| --- | --- | --- | --- | --- | --- | --- | --- | --- | --- | --- | --- | --- | --- | --- | --- | --- | --- | --- | --- | --- | --- | --- | --- | --- | --- | --- | --- | --- | --- | --- | --- | --- | --- | --- | --- | --- |
|  | ppm | ppm | ppm | ppm | ppm | ppm | ppm | ppb | ppb | ppb | ppb | ppb | ppb | ppb | ppb | ppb | ppb | ppb | ppb | ppb | ppb | ppb | ppb | ppb | ppb | ppb | ppb | ppb | ppb | ppb | ppb | ppb | ppb | ppb | ppb | ppb |
| TNB01-AU | 485.8 | 44.88 | 251.4 | 66.43 | 7.761 | 48.30 | 73.66 | 2066 | 7975 | 18701 | 15637 | 31728 | 100607 | 28067 | 19645 | 1281 | <200 | 4007 | 40393 | 84925 | 9769 | 50778 | 7352 | 1545 | 7694 | 1016 | 5564 | 1027 | 3066 | 409 | 2615 | 393 | 1565 | 18246 | 14299 | 2924 |
| TNB01-AL | 580.1 | 36.12 | 263.9 | 60.32 | 8.067 | 47.99 | 125.2 | 2220 | 8071 | 18577 | 14656 | 34497 | 97064 | 23586 | 16259 | 1103 | <200 | 3503 | 43733 | 88714 | 9960 | 51637 | 7068 | 1532 | 7297 | 909 | 4823 | 867 | 2545 | 328 | 2109 | 321 | 1635 | 17323 | 14066 | 2868 |
| TNB01-BU | 544.5 | 44.44 | 307.7 | 70.17 | 6.606 | 43.58 | 36.27 | 2107 | 7366 | 18410 | 11992 | 33867 | 87261 | 27682 | 24250 | 1229 | <200 | 2599 | 73559 | 144866 | 15825 | 82399 | 9562 | 1786 | 9600 | 1134 | 5675 | 1041 | 3097 | 401 | 2496 | 382 | 2502 | 17769 | 20837 | 2525 |
| TNB01-BL | 616.6 | 45.67 | 307.3 | 75.36 | 7.206 | 44.15 | 184.1 | 2296 | 7936 | 20996 | 14060 | 37073 | 98815 | 28741 | 23287 | 1213 | 1115 | 2584 | 51583 | 105493 | 12047 | 62480 | 8736 | 1816 | 8961 | 1121 | 5903 | 1083 | 3222 | 420 | 2752 | 409 | 2496 | 19472 | 14563 | 2824 |
| TNB01-CU | 499.9 | 36.71 | 255.9 | 73.59 | 8.521 | 54.42 | 82.30 | 2016 | 9226 | 23146 | 17575 | 31719 | 102338 | 28218 | 14257 | 1187 | <200 | 4284 | 42059 | 84899 | 9598 | 50173 | 7042 | 1502 | 7459 | 973 | 5327 | 1018 | 3085 | 411 | 2700 | 402 | 1755 | 18041 | 13081 | 3003 |
| TNB01-CL | 526.4 | 35.25 | 270.1 | 67.14 | 8.331 | 49.20 | 210.8 | 2169 | 8438 | 20036 | 15492 | 32012 | 97461 | 28919 | 16011 | 1117 | <200 | 3643 | 40656 | 82509 | 9317 | 48890 | 6911 | 1476 | 7283 | 974 | 5429 | 1027 | 3092 | 429 | 2804 | 431 | 1291 | 17444 | 13777 | 4660 |
| TNB02-AU | 474.0 | 32.21 | 301.7 | 60.90 | 6.305 | 38.61 | 122.2 | 1916 | 6925 | 17219 | 8500 | 30233 | 84799 | 26736 | 14928 | 989 | <200 | 2184 | 56867 | 112985 | 12377 | 64170 | 8099 | 1616 | 8317 | 1030 | 5318 | 975 | 2872 | 376 | 2371 | 356 | 1690 | 15467 | 18089 | 2319 |
| TNB02-AL | 485.6 | 27.61 | 301.0 | 72.09 | 6.583 | 37.75 | 66.63 | 2075 | 8018 | 19360 | 8966 | 31781 | 88392 | 31099 | 18775 | 1088 | <200 | 2207 | 48561 | 98965 | 11259 | 58797 | 8076 | 1670 | 8430 | 1098 | 5862 | 1103 | 3354 | 442 | 2821 | 433 | 1706 | 15271 | 14646 | 2478 |
| TNB02-BU | 470.6 | 30.78 | 261.5 | 79.13 | 7.035 | 39.52 | 269.6 | 2222 | 10647 | 29174 | 10178 | 33041 | 94110 | 34567 | 41269 | 1851 | 203 | 2404 | 55883 | 115023 | 13043 | 68494 | 9476 | 1819 | 9833 | 1266 | 6828 | 1274 | 3796 | 507 | 3239 | 492 | 4527 | 15935 | 15342 | 2836 |
| TNB02-BL | 500.1 | 29.74 | 271.1 | 98.50 | 8.024 | 46.72 | 166.3 | 2276 | 10728 | 33153 | 12000 | 34053 | 104220 | 34587 | 22688 | 1655 | 201 | 2710 | 55479 | 114181 | 12982 | 67370 | 9218 | 1825 | 9826 | 1270 | 6794 | 1251 | 3756 | 488 | 3107 | 473 | 2313 | 15335 | 16250 | 2747 |
| TNB02-CU | 534.5 | 31.96 | 298.0 | 70.09 | 6.534 | 36.67 | 107.4 | 2138 | 6830 | 15537 | 8961 | 34080 | 89445 | 29504 | 20616 | 1346 | <200 | 2038 | 47122 | 95381 | 10883 | 56351 | 7904 | 1773 | 8272 | 1078 | 5756 | 1072 | 3164 | 418 | 2671 | 405 | 1823 | 16122 | 13262 | 2576 |
| TNB02-CL | 474.6 | 30.22 | 262.8 | 75.40 | 6.827 | 38.18 | 181.9 | 2519 | 7309 | 16238 | 9074 | 32829 | 92665 | 30483 | 17778 | 1308 | <200 | 2218 | 49255 | 101479 | 11528 | 59770 | 8354 | 1725 | 8739 | 1119 | 5956 | 1122 | 3340 | 444 | 2856 | 433 | 1456 | 15119 | 13319 | 2522 |
| TNB03-AU | 397.4 | 46.44 | 227.9 | 59.00 | 7.313 | 45.98 | 51.51 | 2104 | 7729 | 21089 | 22283 | 26418 | 83193 | 19758 | 12674 | 1170 | <200 | 3571 | 41792 | 84435 | 9385 | 48715 | 6384 | 1340 | 6601 | 808 | 4085 | 729 | 2103 | 262 | 1667 | 247 | 1136 | 17309 | 13074 | 2723 |
| TNB03-AL | 401.0 | 83.94 | 234.0 | 66.71 | 7.492 | 60.80 | 60.89 | 2723 | 8588 | 22251 | 33958 | 28049 | 84477 | 21275 | 13526 | 1950 | <200 | 3820 | 38727 | 77352 | 8599 | 45018 | 6228 | 1416 | 6378 | 819 | 4353 | 786 | 2342 | 302 | 1925 | 286 | 884 | 17033 | 12265 | 2978 |
| TNB03-BU | 479.6 | 33.86 | 251 | 60.95 | 7.649 | 41.27 | 122.7 | 1850 | 7860 | 17824 | 18507 | 30671 | 91208 | 25894 | 7881 | 1047 | <200 | 3492 | 43744 | 89765 | 10180 | 53015 | 7452 | 1560 | 7782 | 993 | 5336 | 970 | 2921 | 397 | 2770 | 423 | 289 | 19180 | 14939 | 2944 |
| TNB03-BL | 499.7 | 51.52 | 242.0 | 69.33 | 8.062 | 49.28 | 125.7 | 1647 | 9420 | 23443 | 18985 | 30781 | 91857 | 23314 | 19905 | 1132 | <200 | 4067 | 56405 | 114717 | 12718 | 66619 | 8650 | 1576 | 8733 | 1052 | 5137 | 861 | 2376 | 291 | 1827 | 276 | 1996 | 20728 | 20325 | 3704 |
| TNB03-CU | 500.7 | 13.40 | 220.2 | 51.73 | 5.699 | 39.46 | 87.10 | 1560 | 6066 | 13325 | 25660 | 28845 | 82587 | 17237 | 4247 | 1127 | <200 | 3214 | 41888 | 84530 | 9271 | 48738 | 6207 | 1386 | 6274 | 763 | 3693 | 642 | 1784 | 213 | 1354 | 200 | 206 | 20195 | 15070 | 2568 |
| TNB03-CL | 484.9 | 21.11 | 224 | 62.03 | 6.909 | 46.22 | 91.86 | 1702 | 8721 | 17519 | 26986 | 30311 | 93148 | 22499 | 14930 | 1278 | <200 | 3584 | 52082 | 106369 | 11919 | 61927 | 8231 | 1512 | 8359 | 1006 | 4891 | 846 | 2360 | 285 | 1752 | 264 | 1449 | 20004 | 17990 | 3539 |
| TNB04-AU | 487.4 | 71.87 | 196.2 | 79.01 | 9.266 | 84.91 | 61.96 | 3016 | 10994 | 28999 | 55521 | 31761 | 126635 | 19558 | 17995 | 2371 | 211 | 5922 | 45524 | 91558 | 10214 | 53427 | 7010 | 1384 | 7155 | 868 | 4205 | 727 | 2021 | 247 | 1565 | 234 | 1621 | 21181 | 16067 | 4094 |
| TNB04-AL | 505.0 | 44.96 | 192.0 | 90.12 | 10.62 | 97.37 | 187.1 | 3115 | 13391 | 32501 | 74213 | 33495 | 143233 | 29779 | 28055 | 2430 | 200 | 7017 | 47887 | 95885 | 10668 | 55629 | 7505 | 1443 | 7895 | 1025 | 5561 | 1051 | 3069 | 398 | 2482 | 375 | 2833 | 21404 | 17290 | 4991 |
| TNB04-BU | 446.0 | 81.41 | 280.0 | 115.4 | 11.01 | 71.18 | 142.2 | 2966 | 14905 | 40918 | 23419 | 36312 | 121121 | 40133 | 15620 | 2862 | 228 | 4134 | 67727 | 133628 | 14896 | 77843 | 10352 | 2056 | 11066 | 1452 | 7869 | 1466 | 4363 | 573 | 3668 | 540 | 1570 | 18184 | 17299 | 6221 |
| TNB04-BL | 452.7 | 65.38 | 269.5 | 139.1 | 10.80 | 76.96 | 300.0 | 3029 | 15844 | 39318 | 27818 | 36954 | 128152 | 42249 | 22013 | 3338 | 229 | 4599 | 76250 | 152026 | 16828 | 88269 | 11561 | 2160 | 12308 | 1596 | 8348 | 1526 | 4497 | 584 | 3613 | 543 | 1143 | 17719 | 18787 | 4769 |
| TNB04-CU | 490.5 | 100.65 | 236.2 | 92.46 | 13.36 | 101.8 | 31.18 | 2298 | 16845 | 43252 | 75097 | 35303 | 146889 | 28692 | 14642 | 1896 | 221 | 6771 | 55589 | 111947 | 12596 | 64975 | 8840 | 1666 | 9171 | 1136 | 5862 | 1023 | 2962 | 371 | 2344 | 353 | 854 | 21940 | 20234 | 6136 |
| TNB04-CL | 511.0 | 71.30 | 233.7 | 90.57 | 13.79 | 104.1 | 53.77 | 2310 | 17456 | 42678 | 77850 | 35905 | 146680 | 31836 | 20350 | 2007 | <200 | 6755 | 63822 | 127169 | 14423 | 75108 | 10003 | 1769 | 10312 | 1265 | 6399 | 1140 | 3226 | 404 | 2551 | 380 | 1334 | 22093 | 22282 | 6026 |
| TNB05-AU | 501.9 | 35.32 | 222.5 | 39.36 | 6.086 | 37.41 | 20.08 | 1407 | 6470 | 13775 | 18125 | 29435 | 93695 | 18145 | 8448 | 975 | 227 | 4495 | 45630 | 92144 | 10097 | 52855 | 6990 | 1340 | 7091 | 856 | 4131 | 687 | 1827 | 216 | 1284 | 189 | 657 | 24321 | 16305 | 3574 |
| TNB05-AL | 508.2 | 50.29 | 227.1 | 45.05 | 5.232 | 41.60 | 14.70 | 1695 | 8029 | 21492 | 23919 | 29409 | 89098 | 14958 | 5235 | 566 | 4216 | 4641 | 28050 | 56388 | 6308 | 32799 | 4521 | 1304 | 4561 | 576 | 2993 | 547 | 1603 | 201 | 1286 | 190 | 455 | 21388 | 9838 | 2584 |
| TNB05-BU | 494.9 | 28.30 | 224.4 | 41.33 | 7.186 | 42.33 | 72.76 | 1611 | 7458 | 20274 | 21093 | 30876 | 96896 | 14943 | 14070 | 1057 | <200 | 4862 | 31280 | 63763 | 7165 | 37160 | 5095 | 1325 | 4988 | 619 | 3060 | 533 | 1515 | 192 | 1221 | 188 | 1509 | 22990 | 10427 | 3006 |
| TNB05-BL | 450.1 | 43.99 | 220.7 | 43.54 | 6.905 | 41.01 | 29.30 | 1676 | 7187 | 19891 | 22630 | 28006 | 89196 | 17324 | 10059 | 997 | <200 | 4719 | 38625 | 78873 | 8723 | 45406 | 6086 | 1350 | 6027 | 734 | 3629 | 627 | 1813 | 227 | 1476 | 225 | 751 | 20424 | 13420 | 3591 |
| TNB05-CU | 456.3 | 41.71 | 219.8 | 46.98 | 6.549 | 39.89 | 95.92 | 1661 | 8038 | 20360 | 18790 | 28510 | 91504 | 15605 | 15907 | 946 | <200 | 4289 | 31511 | 63785 | 7162 | 37136 | 5040 | 1299 | 5043 | 639 | 3170 | 562 | 1644 | 208 | 1317 | 197 | 1751 | 20891 | 11053 | 2770 |
| TNB05-CL | 564.7 | 47.25 | 209.0 | 44.83 | 7.048 | 45.42 | 41.62 | 1373 | 7548 | 24445 | 19646 | 31109 | 105729 | 14165 | 4958 | 927 | <200 | 6054 | 36336 | 72101 | 7968 | 41909 | 5556 | 1245 | 5591 | 658 | 3139 | 518 | 1430 | 168 | 1027 | 153 | 220 | 24355 | 13523 | 3197 |
| TNB06-AU | 437.2 | 30.98 | 213.6 | 42.14 | 5.280 | 30.18 | 82.23 | 1753 | 13054 | 19524 | 22146 | 25817 | 72224 | 35015 | 9851 | 624 | <200 | 2753 | 36888 | 78816 | 9242 | 47981 | 7290 | 1456 | 7642 | 1059 | 6030 | 1206 | 3753 | 520 | 3377 | 517 | 580 | 19041 | 13653 | 2792 |
| TNB06-AL | 452.3 | 25.60 | 212.5 | 33.43 | 4.734 | 28.23 | 76.41 | 1384 | 7686 | 19901 | 11819 | 26281 | 78212 | 17238 | 5312 | 558 | <200 | 2616 | 42658 | 81910 | 8636 | 45601 | 5467 | 1262 | 5691 | 689 | 3434 | 613 | 1774 | 221 | 1430 | 214 | 461 | 19616 | 16978 | 2375 |
| TNB06-BU | 423.3 | 33.49 | 205.3 | 32.23 | 5.674 | 35.25 | 77.32 | 1427 | 5634 | 15414 | 11604 | 24873 | 70989 | 26804 | 10807 | 620 | <200 | 2495 | 57316 | 118723 | 13444 | 69680 | 9580 | 1413 | 9472 | 1151 | 5552 | 955 | 2686 | 329 | 2087 | 309 | 539 | 18982 | 24172 | 3951 |
| TNB06-BL | 431.7 | 20.75 | 209.8 | 24.86 | 4.209 | 24.27 | 70.52 | 1405 | 4685 | 10823 | 9876 | 25621 | 73067 | 28833 | 8981 | 542 | <200 | 2461 | 33433 | 69159 | 7726 | 40398 | 5635 | 1241 | 6008 | 826 | 4822 | 992 | 3041 | 425 | 2664 | 398 | 983 | 19855 | 13055 | 2785 |
| TNB06-CU | 432.5 | 7.95 | 192.1 | 19.76 | 3.202 | 19.62 | 47.10 | 1275 | 3954 | 9554 | 8684 | 24478 | 68672 | 9441 | 5214 | 457 | <200 | 1999 | 26727 | 53230 | 5780 | 30054 | 3706 | 1072 | 3571 | 418 | 2004 | 340 | 960 | 117 | 754 | 114 | 647 | 18394 | 8379 | 1178 |
| TNB06-CL | 487.4 | 22.19 | 210.9 | 27.89 | 4.457 | 25.59 | 61.73 | 1440 | 4429 | 9927 | 11435 | 27117 | 77043 | 14767 | 8882 | 660 | <200 | 2614 | 28562 | 58077 | 6612 | 34037 | 4674 | 1264 | 4676 | 603 | 2959 | 530 | 1501 | 190 | 1201 | 183 | 911 | 19090 | 10421 | 1766 |
| TNB07-AU | 423.6 | 12.57 | 301.7 | 36.25 | 5.320 | 32.67 | 66.48 | 1734 | 5863 | 12982 | 7131 | 27672 | 73814 | 20783 | 6446 | 640 | <200 | 2319 | 39864 | 81492 | 9209 | 47728 | 6414 | 1420 | 6541 | 799 | 4067 | 736 | 2162 | 278 | 1737 | 253 | 418 | 16888 | 13261 | 2025 |
| TNB07-AL | 437.2 | 10.98 | 316.4 | 35.79 | 5.635 | 32.50 | 104.64 | 1839 | 5556 | 10637 | 7914 | 27978 | 71911 | 29922 | 4293 | 680 | <200 | 2116 | 51117 | 107034 | 12245 | 63961 | 8807 | 1650 | 9099 | 1131 | 5779 | 1034 | 3105 | 409 | 2575 | 388 | 318 | 16293 | 19621 | 2676 |
| TNB07-BU | 433.1 | 67.63 | 211.0 | 68.53 | 9.172 | 55.03 | 140.67 | 2206 | 12316 | 32823 | 21271 | 31697 | 114656 | 29551 | 7239 | 1580 | <200 | 5038 | 54667 | 109861 | 12160 | 63232 | 8440 | 1684 | 8861 | 1137 | 5799 | 1057 | 3085 | 401 | 2536 | 384 | 498 | 16963 | 15832 | 3070 |
| TNB07-BL | 447.0 | 58.66 | 218.4 | 79.83 | 10.18 | 61.29 | 157.76 | 2122 | 13252 | 35121 | 23711 | 33147 | 124802 | 31199 | 14328 | 603 | 208 | 5594 | 55412 | 112195 | 12653 | 66342 | 9100 | 1719 | 9418 | 1218 | 6254 | 1126 | 3230 | 412 | 2656 | 386 | 1561 | 17295 | 16515 | 3362 |
| TNB07-CU | 438.1 | 20.86 | 229.5 | 48.90 | 7.220 | 44.52 | 98.79 | 1886 | 8786 | 24532 | 14269 | 32336 | 98227 | 23076 | 9732 | 1082 | <200 | 3570 | 46325 | 92764 | 10419 | 54424 | 7222 | 1511 | 7378 | 894 | 4542 | 803 | 2400 | 307 | 1960 | 294 | 629 | 18365 | 14880 | 2801 |
| TNB07-CL | 426.5 | 26.52 | 251.2 | 52.48 | 6.831 | 40.42 | 129.27 | 1952 | 8457 | 25076 | 13790 | 29763 | 96664 | 25149 | 5144 | 1032 | <200 | 3419 | 38646 | 78436 | 8925 | 46723 | 6646 | 1589 | 6918 | 918 | 4774 | 888 | 2644 | 340 | 2182 | 329 | 249 | 17914 | 12316 | 2733 |
| Min | 397.4 | 7.95 | 192.0 | 19.76 | 3.202 | 19.62 | 14.70 | 1275 | 3954 | 9554 | 7131 | 24478 | 68672 | 9441 | 4247 | 457 | 200 | 1999 | 26727 | 53230 | 5780 | 30054 | 3706 | 1072 | 3571 | 418 | 2004 | 340 | 960 | 117 | 754 | 114 | 206 | 15119 | 8379 | 1178 |
| Max | 616.6 | 100.65 | 316.4 | 139.09 | 13.790 | 104.14 | 300.05 | 3115 | 17456 | 43252 | 77850 | 37073 | 146889 | 42249 | 41269 | 3338 | 4216 | 7017 | 76250 | 152026 | 16828 | 88269 | 11561 | 2160 | 12308 | 1596 | 8348 | 1526 | 4497 | 584 | 3668 | 543 | 4527 | 24355 | 24172 | 6221 |
| Average | 478.4 | 40.39 | 244.2 | 62.11 | 7.398 | 48.14 | 103.09 | 2004 | 8945 | 22386 | 21791 | 30853 | 96732 | 25270 | 14203 | 1251 | 660 | 3747 | 46658 | 94466 | 10590 | 55191 | 7440 | 1536 | 7674 | 969 | 5027 | 914 | 2681 | 347 | 2210 | 332 | 1267 | 18847 | 15257 | 3219 |
| STD | 46.8 | 20.71 | 35.3 | 24.54 | 2.223 | 19.69 | 63.54 | 478 | 3261 | 8845 | 17466 | 3335 | 20099 | 7365 | 7581 | 639 | 1210 | 1397 | 11529 | 22857 | 2552 | 13324 | 1728 | 228 | 1859 | 243 | 1354 | 263 | 815 | 114 | 733 | 111 | 859 | 2365 | 3395 | 1098 |
