# Supplementary material for: Highly Heterogeneous Soil Bacterial Communities around Terra Nova Bay of Northern Victoria Land, Antarctica
Source: PLoS One. 2015 Mar 23;10(3):e0119966. doi: 10.1371/journal.pone.0119966 (PMC4370865; doi:10.1371/journal.pone.0119966)
Supplement: S4 Table — (DOCX) [file pone.0119966.s007.docx]

**S4 Table. Summary of 454 data analysis and diversity estimates**

| **Samples** | **Pre-processed reads*** | **Quality-filtered reads** | **OTUs**** | **Shannon**** | **InvSimpson**** | **Phylogenetic diversity**** |
| --- | --- | --- | --- | --- | --- | --- |
| **TNB01-AU** | 1688 | 1314 | 217 | 4.78 | 63.45 | 0.142 |
| **TNB01-AL** | 1458 | 1083 | 218 | 4.85 | 84.72 | 0.140 |
| **TNB01-BU** | 1614 | 1281 | 283 | 4.94 | 39.53 | 0.134 |
| **TNB01-BL** | 1276 | 1056 | 219 | 4.52 | 28.21 | 0.133 |
| **TNB01-CU** | 1838 | 1492 | 242 | 4.65 | 26.07 | 0.135 |
| **TNB01-CL** | 1819 | 1349 | 252 | 4.96 | 77.42 | 0.149 |
| **TNB02-AU** | 1206 | 909 | 246 | 4.99 | 81.12 | 0.164 |
| **TNB02-AL** | 1695 | 1321 | 223 | 4.81 | 61.60 | 0.140 |
| **TNB02-BU** | 1910 | 1460 | **312** | **5.47** | **245.79** | 0.170 |
| **TNB02-BL** | 1667 | 1282 | 196 | 4.55 | 45.54 | 0.142 |
| **TNB02-CU** | 1510 | 1148 | 254 | 5.03 | 76.13 | 0.170 |
| **TNB02-CL** | 1351 | 1033 | 232 | 4.90 | 71.00 | 0.143 |
| **TNB03-AU** | 1564 | 1154 | 197 | 4.38 | 30.85 | 0.177 |
| **TNB03-AL** | 1631 | 1174 | 234 | 4.87 | 70.90 | 0.162 |
| **TNB03-BU** | 1375 | 1071 | 203 | 4.70 | 54.25 | 0.138 |
| **TNB03-BL** | 1841 | 1420 | 253 | 5.12 | 131.42 | 0.162 |
| **TNB03-CU** | 1418 | 993 | 198 | 4.61 | 51.26 | 0.158 |
| **TNB03-CL** | 1484 | 1087 | 234 | 5.06 | 126.69 | 0.152 |
| **TNB04-AU** | 1603 | 1243 | 279 | 5.17 | 100.50 | 0.149 |
| **TNB04-AL** | 1823 | 1429 | 277 | 5.20 | 126.20 | 0.155 |
| **TNB04-BU** | 1332 | 998 | 276 | 5.05 | 74.27 | 0.142 |
| **TNB04-BL** | 1332 | 909 | 281 | 5.29 | 167.84 | 0.151 |
| **TNB04-CU** | 1592 | 1206 | 294 | 5.31 | 158.01 | 0.156 |
| **TNB04-CL** | 1489 | 1153 | 187 | 4.31 | 30.86 | 0.130 |
| **TNB05-AU** | 1736 | 1328 | 227 | 4.81 | 68.24 | 0.132 |
| **TNB05-AL** | 1583 | 1270 | 225 | 4.88 | 68.31 | 0.128 |
| **TNB05-BU** | 2558 | 2104 | 225 | 4.54 | 27.64 | 0.135 |
| **TNB05-BL** | 1729 | 1367 | 258 | 4.98 | 78.78 | 0.111 |
| **TNB05-CU** | 1662 | 1284 | 221 | 4.43 | 17.78 | 0.134 |
| **TNB05-CL** | 2187 | 1847 | 174 | 4.18 | 21.72 | 0.124 |
| **TNB06-AU** | 1227 | 953 | 280 | 5.25 | 148.82 | 0.136 |
| **TNB06-AL** | 1385 | 1061 | 232 | 4.80 | 57.75 | 0.128 |
| **TNB06-BU** | 1951 | 1499 | 294 | 5.31 | 165.58 | 0.143 |
| **TNB06-BL** | 1799 | 1313 | 268 | 5.10 | 91.19 | 0.148 |
| **TNB06-CU** | 848 | 628 | 264 | 5.34 | 226.82 | 0.132 |
| **TNB06-CL** | 1438 | 1095 | 262 | 5.21 | 158.64 | 0.137 |
| **TNB07-AU** | 1831 | 1337 | **306** | **5.36** | **162.98** | **0.178** |
| **TNB07-AL** | 1412 | 952 | 258 | 5.18 | 139.04 | 0.152 |
| **TNB07-BU** | 1812 | 1549 | **116** | **3.25** | **7.79** | **0.104** |
| **TNB07-BL** | 1367 | 1176 | **105** | **3.27** | **8.99** | **0.101** |
| **TNB07-CU** | 1894 | 1488 | 267 | 5.04 | 79.26 | 0.142 |
| **TNB07-CL** | 1521 | 1221 | 294 | 5.26 | 122.36 | 0.154 |

* Raw reads were initially trimmed by PyroTrimmer

** Diversity indices were estimated using randomly generated subset (n=628) per sample
